# The functionally conserved human lncRNA motif GULF lowers glucose and lipid levels in obese mice

**DOI:** 10.1172/JCI186355

**Published:** 2025-09-16

**Authors:** Zhe Li, Sunmi Seok, Chengfei Jiang, Ping Li, Yonghe Ma, Hang Sun, Haiming Cao

**Affiliations:** Cardiovascular Branch, National Heart, Lung, and Blood Institute, NIH, Bethesda, Maryland, USA.

**Keywords:** Metabolism, Therapeutics, Diabetes, Drug therapy, Noncoding RNAs

## Abstract

Growing evidence links human long noncoding RNAs (lncRNAs) to metabolic disease pathogenesis, yet no FDA-approved drugs target human lncRNAs. Most human lncRNAs lack conservation in other mammals, complicating efforts to define their roles and identify therapeutic targets. Here, we leveraged the concept of functionally conserved lncRNAs (FCLs) — lncRNAs that share function despite no sequence similarity — to develop a framework for identifying human lncRNAs as therapeutic targets for metabolic disorders. We used expression quantitative trait loci mapping and functional conservation analyses to pinpoint human lncRNAs influenced by disease-associated SNPs and with potential functionally conserved mouse equivalents. We identified human and mouse GULLs (glucose and lipid lowering), which regulate glucose and lipid metabolism by binding CRTC2, thereby modulating gluconeogenic genes via CREB and lipogenic genes via SREBP1. Despite their lack of sequence similarity, both lncRNAs demonstrated similar metabolic effects in obese mice, with more pronounced benefits from long-term activation. To identify druggable sites, we mapped GULLs’ binding motifs to CRTC2 (termed GULFs). Standalone human GULF, an RNA oligomer resembling FDA-approved siRNAs, significantly improved glucose and lipid levels in obese mice. This framework highlights functionally conserved human lncRNAs as promising therapeutic targets, exemplified by GULLs’ potential as a glucose- and lipid-lowering therapeutic.

## Abstract 2

Functionally conserved lncRNAs, like GULLs, regulate metabolism via specific binding motifs, highlighting their therapeutic potential as RNA-based treatments for metabolic diseases.

## Graphical abstract

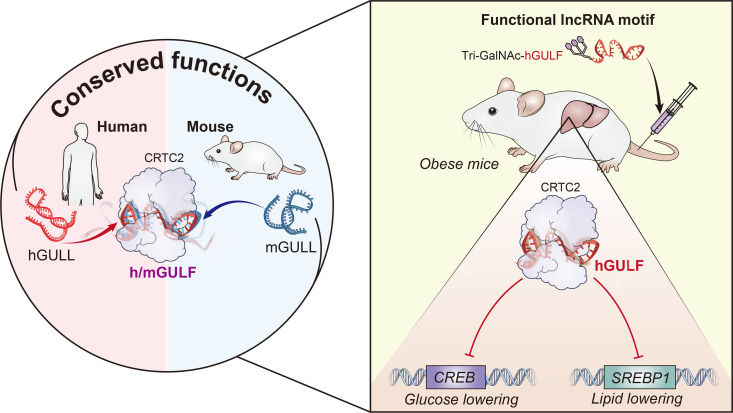

## Introduction

With the FDA approval of multiple siRNA drugs and the ongoing clinical trials of mRNA-based drug candidates for metabolic disorders (1), RNA therapeutics have emerged as effective treatment options for metabolic diseases. However, efforts to develop drugs targeting long noncoding RNAs (lncRNAs) for these diseases have not been successful. Human lncRNAs constitute the largest transcript class of human genome (2). Unlike protein-coding genes, most human lncRNAs are not conserved, even among mammals, making drug development particularly challenging. First, the limited conservation of human lncRNAs poses a major challenge in identifying those with strong pathophysiological potential as drug targets. Genes conferring organismal fitness are typically conserved across species, making conservation a key criterion for functional importance (3). The vast number of human lncRNA genes — 100,000 in the most inclusive databases, 5 times that of protein-coding genes (4) — further complicates this. It remains unclear what fraction of these lncRNAs are functional. Second and most critically, the lack of conservation complicates the experimental definition of their pathophysiological roles. Functional analyses of genes in physiological contexts are essential for clinical translation, typically achieved through homolog studies in genetic mouse models. Although we have previously utilized a liver-specific humanized mouse model to study the in vivo function of nonconserved human lncRNAs (5, 6), we recognize the limitations of this model in providing comprehensive insights into their physiological roles. Third, sequence divergence among species makes it challenging to identify key functional elements of lncRNAs as therapeutic targets. Sequence alignment, a powerful tool for defining functional domains of proteins or miRNA seed sequences (7), is ineffective for nonconserved human lncRNAs. Additionally, resolving the 3D structure of long RNAs at high resolution remains a significant challenge. The lack of effective tools to identify specific functional sites on human lncRNAs poses a daunting task for drug development.

Although most human lncRNAs are considered nonconserved based on their sequences, recent studies, including ours, have demonstrated that some lncRNAs in different species share no sequence similarity but similar function — a phenomenon termed functionally conserved lncRNAs (FCLs) (8, 9). Utilizing this concept, we developed a framework to identify and characterize human lncRNA drug candidates. We performed expression quantitative trait loci (eQTL) mapping and functional conservation analysis to identify disease-associated human lncRNAs with significant functional potential in metabolic physiology. We verified that a pair of such lncRNAs, human and mouse GULLs (glucose and lipid lowering), share similar functions in regulating glucose and lipid metabolism. Mechanistically, both human and mouse GULLs bind to CRTC2 to regulate gluconeogenic genes via CREB and lipogenic genes via SREBP1. Additionally, GULL expression substantially ameliorated glucose and lipid metabolism defects in obese mice, and the effects became more pronounced with longer term activation. Finally, we demonstrated that the binding motifs of human and mouse GULLs to CRTC2 have potent lipid- and glucose-lowering effects in obese mice. Taken together, our findings on the conserved binding motifs of human and mouse GULLs to CRTC2, along with their demonstrated lipid- and glucose-lowering effects in obese mice, support the development of a framework to select human lncRNA drug candidates and identify functionally conserved therapeutic lncRNA motifs.

## Results

### Identification of GULL, a metabolic disease–associated human lncRNA that has a functionally conserved equivalent in mice.

**Figure 1:**
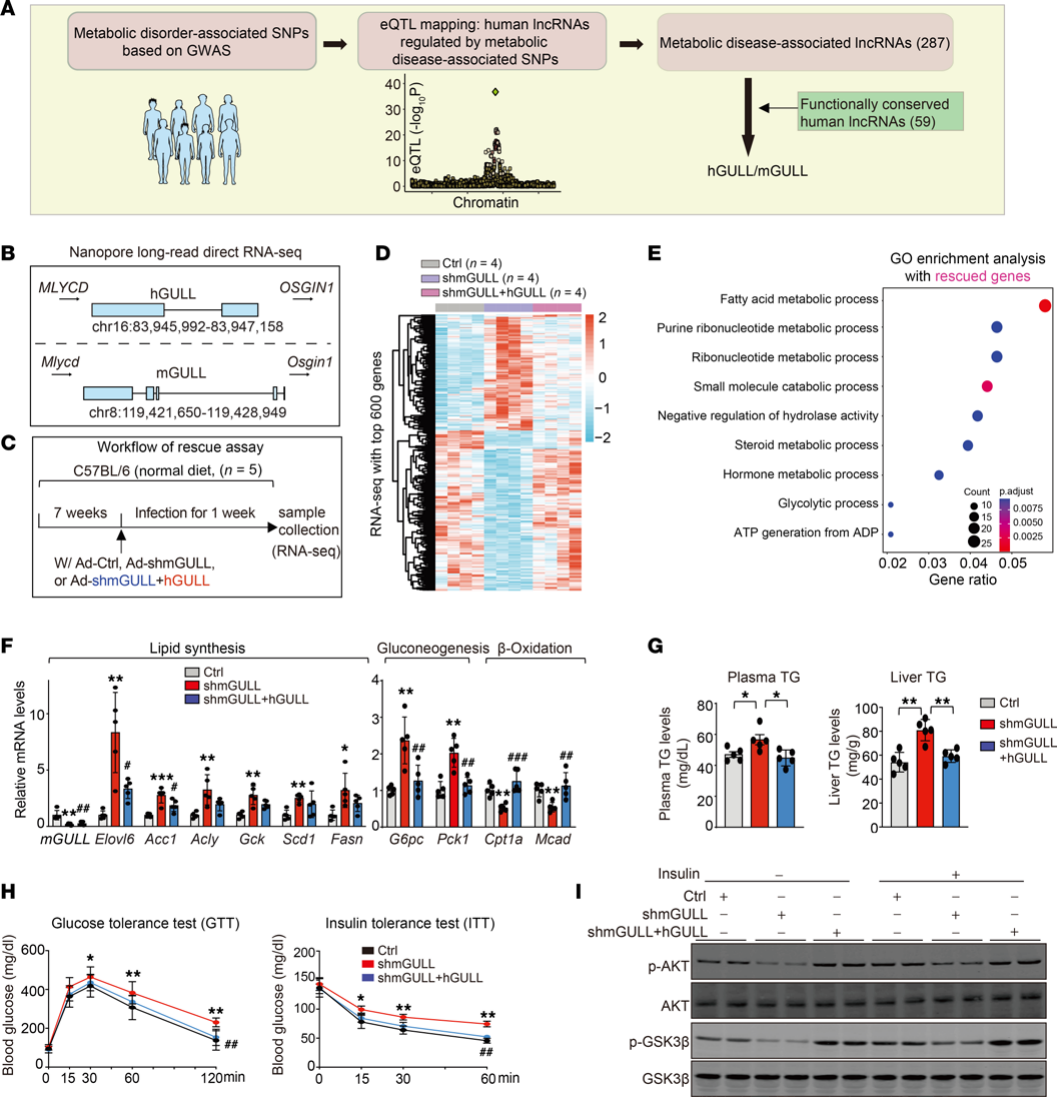
Identification of h/mGULLs as a pair of functionally conserved lncRNAs closely related to metabolic disorders. (**A**) Flowchart for screening the functional conserved lncRNA candidates that are closely associated with metabolic disorders with human GWAS data and nanopore sequencing data (GSE224278). hGULL/mGULL was selected for further study. (**B**) Graphical representation of location and details of hGULL/mGULL on human or mouse chromosomes from Integrative Genomics Viewer and UCSC Genome Browser (human GRCh38/hg38 and mouse GRCm39/mm39). (**C**) Graphical representation of the rescue experiment. Mice were injected with 3 groups of adenoviruses: Ad-Ctrl (Lac+pAdV5), mGULL knockdown (Ad-shmGULL+pAdV5), or rescue group (Ad-shmGULL+Ad-hGULL). (**D**) Heatmap showing the top 600 significantly expressed genes in the mGULL knockdown and mGULL knockdown with hGULL overexpression rescue groups. (**E**) Gene Ontology (GO) enrichment analysis focused on the biological processes that can be rescued, summarized based on |normalized enrichment score| > 1.5, *P* < 0.01, and adjusted *P* value (FDR) < 0.1 from gene set enrichment analysis. The differentially expressed genes were defined as |log_2_(fold change)| > 0.5 and *P* < 0.05. (**F**) Quantitative reverse transcriptase PCR (qRT-PCR) analysis of mRNA levels of lipid synthesis genes (*Elovl6*, *Acc1*, *Acly*, *Gck*, *Scd1*, and *Fasn*), gluconeogenesis genes (*G6pc* and *Pck1*), and β-oxidation genes (*Cpt1a* and *Mcad*) in rescue assay. *Comparison between Ad-Ctrl and Ad-shmGULL groups; ^#^comparison between Ad-shmGULL and Ad-shmGULL+hGULL groups. (**G** and **H**) Plasma/liver TG level, GTT, and ITT were determined in the rescue assay. *Comparison between Ad-Ctrl and Ad-shmGULL groups; ^#^comparison between Ad-shmGULL and Ad-shmGULL+hGULL groups. (**I**) Immunoblot assay determined the protein levels of p-AKT, AKT, p-GSK3β, and GSK3β in the rescue assay with or without insulin treatment. Data are shown as mean ± SD, 1-way ANOVA, in **F**–**H**. *P < 0.05, **P < 0.01, ***P < 0.001; ^#^*P* < 0.05, ^##^*P* < 0.01, ^###^*P* < 0.001.

Since most human lncRNAs are not conserved, confidently identifying those that play important roles in metabolic physiology and can serve as therapeutic targets remains a significant challenge. However, recent reports, including our own work, suggest that some lncRNAs across multiple species share conserved function despite their lack of sequence similarity, a phenomenon termed FCLs (8, 9). Leveraging this concept, we have developed a 2-step approach to select human lncRNAs genetically associated with metabolic diseases that also have functionally conserved counterparts in mice (Figure 1A).

First, we performed eQTL mapping to identify human lncRNAs whose expressions are regulated by single nucleotide polymorphisms (SNPs) associated with metabolic disorders. To do this, we extracted all SNPs associated with metabolic disorders from the NIH GWAS Catalog database (Supplemental Table 1; supplemental material available online with this article; https://doi.org/10.1172/JCI186355DS1), then used a novel annotation of human liver lncRNAs to identify those expressed in the liver (10). We then performed eQTL analysis using Genotype-Tissue Expression Project (GTEx) human liver gene expression data, identifying 287 lncRNAs whose expressions are affected by SNPs linked to metabolic diseases (Supplemental Table 2). Second, we cross-referenced these lncRNAs against a database of functionally conserved lncRNAs between humans and mice that we recently established (9). We selected these FCLs based on criteria of being syntenic, similarly regulated, and correlated to the same metabolic function. Ultimately, we identified 7 human lncRNAs that are genetically associated with metabolic diseases and have functionally conserved equivalents in mice (Figure 1A and Supplemental Table 3).

Careful definition of the pathophysiological function of a potential drug target gene is crucial to understand its disease relevance. For human protein-coding genes, this is often achieved by studying their homologs in mice or other animal models. However, as most human lncRNAs are not conserved, it remains challenging to experimentally define their physiological roles and mechanisms of action within a pathophysiological context because of the lack of suitable models. The 7 human lncRNAs we identified all have potential functional equivalents in mice, allowing their function to be studied in conventional mouse models. Among the 7 candidates, we selected the only pair of intergenic lncRNAs to streamline downstream functional analysis. We named them human and mouse GULLs (glucose and lipid lowering) on the basis of the function identified in this study (Figure 1A).

The newly discovered human and mouse GULLs (h/mGULLs) reside in the syntenic regions between the coding genes *MLYCD*/*Mlycd* and *OSGIN1*/*Osgin1* in humans and mice, respectively (Figure 1B). Coding potential analysis indicates that both lncRNAs are indeed noncoding transcripts (data not shown). After successfully cloning and sequencing this pair of lncRNAs from both species, we performed in vitro translation assays to verify that they do not encode peptides (Supplemental Figure 1A).

As noted, we identified the human lncRNA, hGULL, through a GWAS-eQTL analysis, in which its locus was associated with HDL-C levels. To further assess its disease relevance, we analyzed HDL-C levels in individuals with high and low hGULL expression using public datasets. The results revealed significantly higher HDL-C levels in individuals with elevated hGULL expression, consistent with our eQTL findings and suggesting a potential linkage between hGULL and HDL-C (Supplemental Figure 1B). To further investigate the role of hGULL in metabolic disease pathogenesis, we examined its levels in patients with type 2 diabetes (T2D) and hypertension. Results showed that the hGULL was significantly decreased in the livers of patients with T2D and hypertension compared with healthy individuals (Supplemental Figure 1, C and D). Furthermore, hGULL expression was markedly reduced in livers of individuals with fatty liver, indicating a negative correlation between hGULL expression and fatty liver disease (Supplemental Figure 1E). We also investigated the expression levels of mGULL in the mouse livers under physiological and pathological conditions. Our results demonstrated that mGULL RNA levels were significantly upregulated during refeeding compared with fasting (Supplemental Figure 1F). Moreover, mGULL expression was markedly reduced in mice fed a high-fat diet compared with wild-type controls (Supplemental Figure 1F). These findings highlight a potential connection between GULL and metabolic diseases.

Another key criterion for selecting functionally conserved lncRNAs in humans and mice was their similar regulation by the same metabolic stimuli (9). We initially identified hGULL/mGULL because RNA-Seq showed that the expression levels of both human and mouse GULL in the chimeric human-mouse livers of humanized mice were increased by treatment with the FXR agonist GW4064; this result was verified by real-time PCR (Supplemental Figure 1G). FXR is a bile acid receptor known to regulate glucose and lipid metabolism, and FXR agonists are currently under clinical trials for metabolic dysfunction–associated steatohepatitis and fibrosis (11). To further understand whether FXR directly regulates the expression level of mGULL, we performed a chromatin immunoprecipitation (ChIP) assay and found that the occupancy of FXR and its coreceptor, RXR, at the promoter of mGULL in mouse liver was significantly increased by GW4064 treatment (Supplemental Figure 1H), indicating that FXR directly activates mGULL transcription in mouse liver.

Given the identification of hGULL and mGULL as potential functionally conserved lncRNAs (FCLs), we conducted a rescue experiment in mice to examine the functional similarities between them. We used shRNAs delivered by adenovirus to knock down mGULL in the mouse liver and subsequently coexpressed hGULL to evaluate its ability to rescue the loss-of-function effect of mGULL (Figure 1C and Supplemental Figure 1I). Since lncRNAs are known to regulate gene expression, we performed RNA-Seq on liver tissues and used global gene expression levels as an indicator to assess the functional similarities between hGULL and mGULL. This experiment revealed that a substantial number of genes whose expression levels were regulated by mGULL knockdown exhibited reversed expression patterns in the rescue group with concurrent expression of hGULL, often returning to control group levels (Figure 1D). Additionally, Gene Ontology enrichment analysis of genes regulated by hGULL/mGULL showed significant enrichment in genes involved in the fatty acid metabolic process (Figure 1E).

To validate the RNA-Seq results and further explore the role of GULL in lipid metabolism, we examined the expression levels of a panel of genes in lipid and glucose metabolic pathways in mGULL-knockdown and rescue mice. We found that the expression levels of lipogenic genes, such as *Elovl6*, *Acc1*, *Acly*, *Gck*, *Scd1*, and *Fasn*, and gluconeogenic genes, such as *G6pc* and *Pck1*, were significantly increased in the livers of mGULL-knockdown mice and were reversed by concurrent expression of hGULL (Figure 1F). Meanwhile, the expression of β-oxidation genes showed the opposite pattern (Figure 1F). To understand whether the altered gene expression led to changes in metabolic response, we examined how hGULL/mGULL affected lipid and glucose metabolism. We found that knockdown of mGULL increased the levels of triglycerides (TGs) in the plasma and liver, and the effects were completely reversed by concurrent expression of hGULL (Figure 1G). Similarly, mGULL knockdown resulted in impaired glucose metabolism as shown by a glucose tolerance test (GTT) and insulin tolerance test (ITT), and the effect was rescued by concurrent hGULL expression (Figure 1H). The impaired glucose metabolism in mGULL-knockdown mice suggests that they might have reduced insulin sensitivity. Indeed, an acute insulin stimulation experiment showed that phosphorylation of Akt and Gsk3β — 2 key proteins in the insulin receptor signaling pathway — was dampened in mGULL-knockdown mice and recovered by concurrent hGULL expression (Figure 1I). Taken together, our data support that human and mouse GULLs are legitimate FCLs and have similar lipid- and glucose-lowering effects in mice.

### Both human and mouse GULLs bind to CRTC2 to regulate lipid and glucose metabolism.

**Figure 2:**
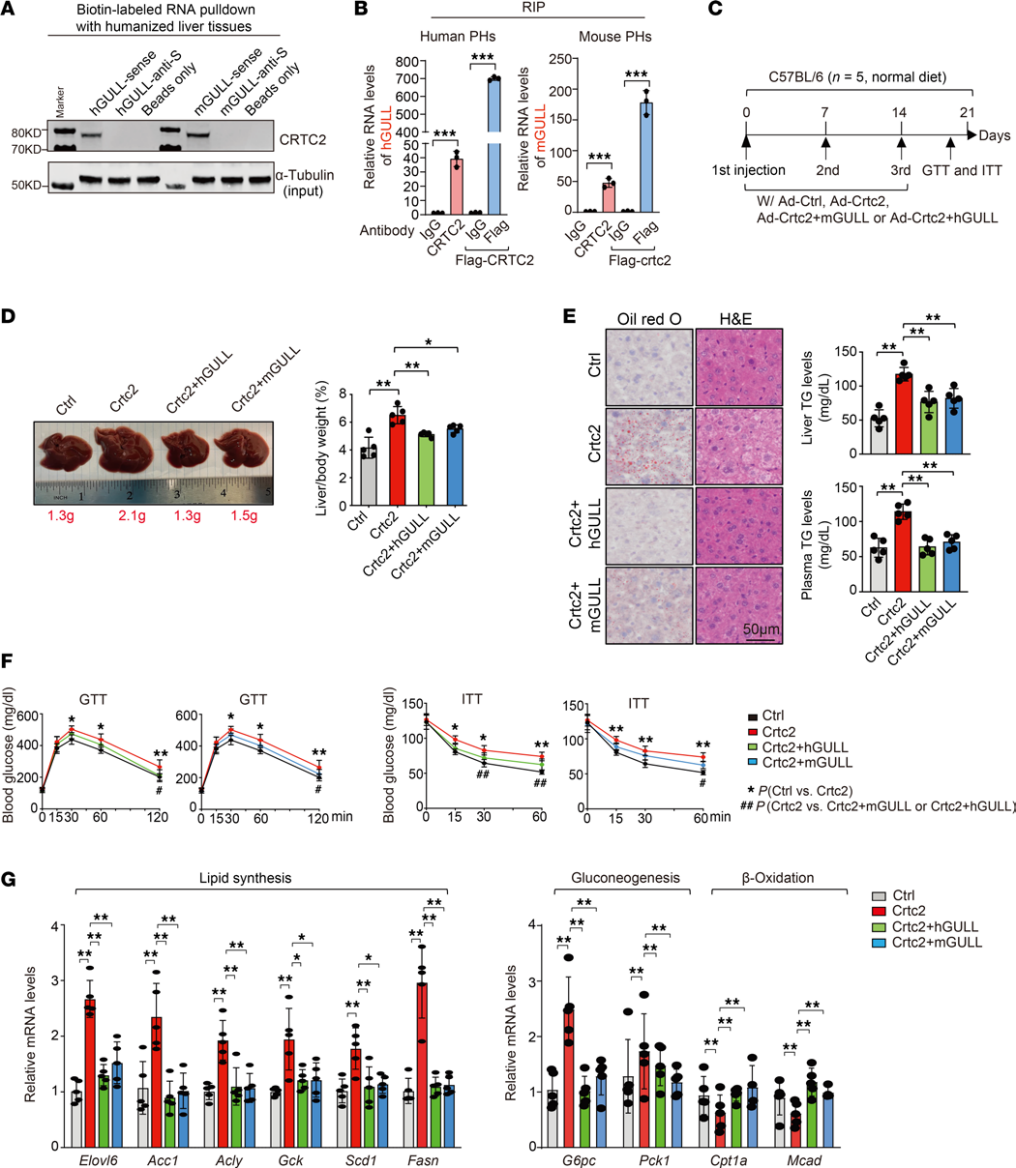
Beneficial effects of hGULL/mGULL on fatty liver are CRTC2 dependent. (**A**) RNA pull-down coupled with immunoblot assay to verify the associations of hGULL/mGULL with CRTC2 in humanized mouse tissues. The antisense and beads-only groups served as negative control. (**B**) RNA immunoprecipitation (RIP) assays were performed with endogenous CRTC2 antibody and FLAG antibodies in human primary hepatocyte (PH) or mouse PH cells, and the coprecipitated RNA was subjected to qRT-PCR with the primers of h/mGULL. ***P < 0.001; data shown as mean ± SD, 2-tailed unpaired Student’s *t* test. (**C**) Graphical representation of mouse model to explore the phenotype and molecular mechanisms between CRTC2 and h/mGULL. Mice were injected with 4 groups of adenoviruses: the Ad-Ctrl group (Lac+pAdV5), Crtc2 overexpression group (Lac+Ad-Crtc2), Crtc2 overexpression with hGULL knockdown group (Ad-Crtc2+hGULL), and Crtc2 overexpression with mGULL knockdown group (Ad-Crtc2+mGULL). (**D**) Representative images of liver and liver/body weight ratio analysis in the 4 groups, including the Ad-Ctrl, Ad-Crtc2 overexpression, and AdCrtc2+hGULL/mGULL overexpression groups. *P < 0.05, **P < 0.01; data shown as mean ± SD, 1-way ANOVA. (**E**) Representative images of Oil Red O and H&E staining and plasma/liver TG level analysis of the Ad-Ctrl, Ad-Crtc2 overexpression, and AdCrtc2+hGULL/mGULL overexpression groups. **P < 0.01; data shown as mean ± SD, 1-way ANOVA. Scale bar: 50 μm. (**F**) GTT and ITT were determined in the Ad-Ctrl, Ad-Crtc2 overexpression, and Ad-Crtc2+hGULL or mGULL overexpression groups. *Comparison between Ad-Ctrl and Ad-Crtc2 groups; *P < 0.05, **P < 0.01. ^#^Comparison between Ad-Crtc2 and AdCrtc2+hGULL or mGULL groups; ^#^*P* < 0.05, ^##^*P* < 0.01. Data shown as mean ± SD, 1-way ANOVA. (**G**) The mRNA levels of lipid synthesis genes (*Elovl6*, *Acc1*, *Acly*, *Gck*, *Scd1*, and *Fasn*), gluconeogenesis genes (*G6pc* and *Pck1*), and β-oxidation genes (*Cpt1a* and *Mcad*) were quantified in Ad-Ctrl, Ad-Crtc2 overexpression, and AdCrtc2+hGULL or mGULL overexpression groups using quantitative PCR (qPCR). *P < 0.05, **P < 0.01; data shown as mean ± SD, 1-way ANOVA.

**Figure 3:**
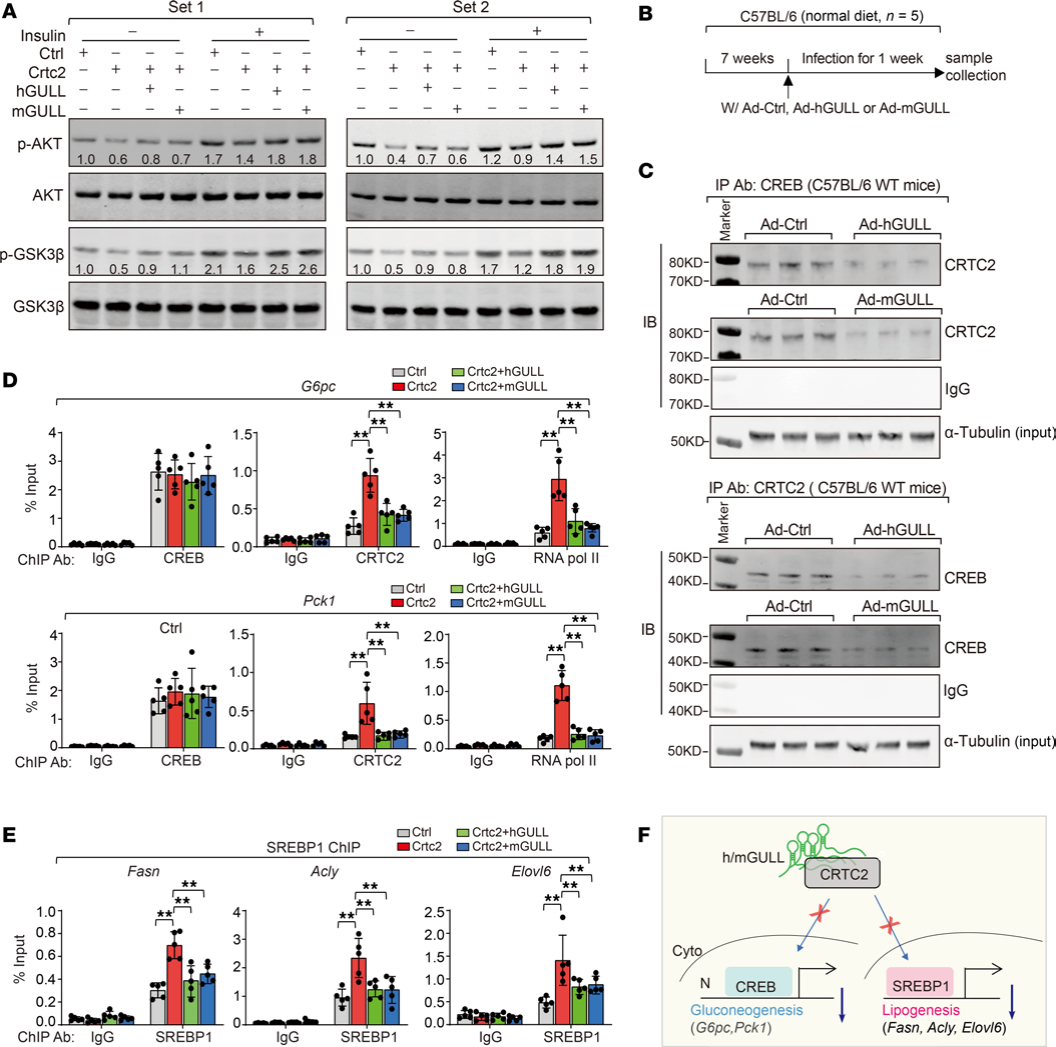
hGULL/mGULL bind to CRTC2 and inhibit its activity in regulating gluconeogenesis and lipogenesis. (**A**) Immunoblotting of p-AKT, AKT, p-GSK3β, and GSK3β in the Ad-Ctrl, Ad-Crtc2 overexpression, and Ad-Crtc2+hGULL or mGULL overexpression groups with or without insulin treatment. (**B**) Graphical representation of mouse model to explore the phenotype and molecular mechanisms of h/mGULL. Mice were injected with 3 groups of adenoviruses: the Ad-Ctrl group (Lac+pAdV5), hGULL overexpression group (Ad-hGULL), or mGULL overexpression group (Ad-mGULL). (**C**) Coimmunoprecipitation to detect the association between CRTC2 and CREB after hGULL/mGULL overexpression. Mice were injected with Ctrl (pAdV5) and hGULL or mGULL adenoviruses and treated with 6 hours of fasting before sacrifice. (**D**) ChIP-qPCR detection of occupancy of CREB or CRTC2 and RNA polymerase II level on *G6pc* and *Pck1* promoter in Ad-Ctrl, Ad-Crtc2 overexpression, and Ad-Crtc2+hGULL or mGULL groups. IgG was used as negative control. **P < 0.01; data shown as mean ± SD, 2-way ANOVA. (**E**) ChIP-qPCR determined SREBP1 occupancy on *Fasn*, *Acly*, or *Elovl6* promoter in the Ad-Ctrl, Ad-Crtc2 overexpression, and Ad-Crtc2+hGULL or mGULL groups. IgG was used as negative control. **P < 0.01; data shown as mean ± SD, 2-way ANOVA. (**F**) Graphical representation of working model of hGULL/mGULL-mediated glucose or lipid lowering. A higher RNA level of hGULL/mGULL specifically binds with CRTC2 and holds it in the cytoplasm, decreasing the occupancy of CREB or SREBP1 at the promoter of gluconeogenesis and lipogenesis genes.

LncRNAs often carry out their biological functions via interaction with specific binding proteins. To identify potential protein-binding partners of hGULL and mGULL, we used biotinylated versions of these RNAs to perform RNA pull-down assays coupled with proteomic analysis, using chimeric human-mouse liver tissues from GW4064-treated humanized mice. We identified 2 proteins, CREB-regulated transcription coactivator 2 (CRTC2) and argininosuccinate synthase 1 (ASS1), that bind to both hGULL and mGULL (Supplemental Table 4). As CRTC2 is a well-known regulator of lipid and glucose metabolism (12), we focused on its role in the function of hGULL/mGULL. Immunoblot assay verified that the sense, but not antisense, of hGULL and mGULL specifically binds to CRTC2 (Figure 2A). In addition, we performed 2 sets of RNA immunoprecipitation (RIP) assays to examine hGULL/mGULL and CRTC2 interaction. First, we immunoprecipitated endogenous CRTC2 in human and mouse primary hepatocytes using CRTC2 antibody. Second, we overexpressed FLAG-tagged CRTC2 or Crtc2 in human and mouse primary hepatocytes, respectively, and then used anti-FLAG antibody to isolate these proteins. Both experiments showed that CRTC2 strongly binds to hGULL and mGULL (Figure 2B), supporting its role in the biological functions of hGULL and mGULL. CRTC2, functioning as a CREB coactivator, plays a crucial role in regulating gluconeogenesis in the liver. Studies show that prolonged activation of CRTC2 during insulin resistance contributes significantly to hyperglycemia (13–15). To determine whether hGULL and mGULL regulate CRTC2 function, we overexpressed Crtc2 to mimic obesity condition (16) and subsequently expressed hGULL or mGULL to examine their functional interaction (Figure 2C). Overexpression of Crtc2 resulted in significantly increased liver size and liver/body weight ratio, indicators of fatty liver. This phenotype was reversed by overexpression of either hGULL or mGULL, suggesting that GULL might inhibit Crtc2 function (Figure 2D). Consistently, overexpression of Crtc2 significantly increased neutral lipid levels in liver tissue and hepatic and plasma TG levels, all of which were reversed by concurrent expression of hGULL or mGULL (Figure 2E). Furthermore, hGULL and mGULL expression rescued impaired glucose metabolism induced by Crtc2 expression, as shown in glucose and insulin tolerance tests (Figure 2F). Crtc2 overexpression also increased the expression levels of lipogenic and gluconeogenic genes, an effect reversed by hGULL or mGULL expression, while β-oxidation gene expression showed the opposite pattern (Figure 2G). Additionally, hGULL and mGULL expression rescued the reduced insulin-stimulated phosphorylation of AKT and GSK3β caused by Crtc2 expression (Figure 3A), further supporting that hGULL and mGULL inhibit CRTC2 function.

To understand the molecular detail of the interaction between hGULL/mGULL and CRTC2, we generated 3 deletion mutants of CRTC2 (Supplemental Figure 2A) to map the key domains required for GULL binding. Results showed that deletion of either the CREB binding domain (CBD) or regulatory domain (REG) of CRTC2 impaired its interaction with hGULL/mGULL. The affinity of hGULL and mGULL toward each domain was similar (Supplemental Figure 2A), suggesting that they might share binding sites on CRTC2. A binding competition assay showed that hGULL and mGULL could effectively compete with each other for interaction with CRTC2 (Supplemental Figure 2B), supporting this claim.

It is well established that CRTC2 acts as a transcription coactivator of CREB to regulate the transcription of gluconeogenic genes during fasting and glycemic control in both physiological and pathological conditions (17, 18). As the CBD of CRTC2 mediates its interaction with CREB (19, 20), we examined whether hGULL/mGULL affects the CREB-CRTC2 interaction. Overexpression of hGULL or mGULL significantly decreased the CREB-CRTC2 interaction in mouse livers (Figure 3, B and C). Consistent with these results, a ChIP assay revealed that overexpression of Crtc2 increased the occupancy of Crtc2 and RNA polymerase II at the promoter regions of gluconeogenesis genes (*G6pc* and *Pck1*), and this occupancy was decreased by hGULL or mGULL overexpression (Figure 3D). Crtc2 is also known to coordinate the actions of downstream effectors such as Sec23A and Sec31A to regulate the processing of SREBP1 maturation and nuclear translocation (21). We performed an SREBP1 ChIP assay and found that hGULL or mGULL overexpression significantly decreased SREBP1 occupancy at the promoter regions of lipogenic genes such as *Fasn*, *Acly*, and *Elovl6* (Figure 3E). Taken together, these data demonstrate that hGULL and mGULL exhibit potent lipid- and glucose-lowering effects by specific binding to CRTC2 and modulation of the function of its downstream effectors (Figure 3F).

### Human and mouse GULLs ameliorate obesity-induced metabolic abnormalities in a CRTC2-dependent manner.

**Figure 4:**
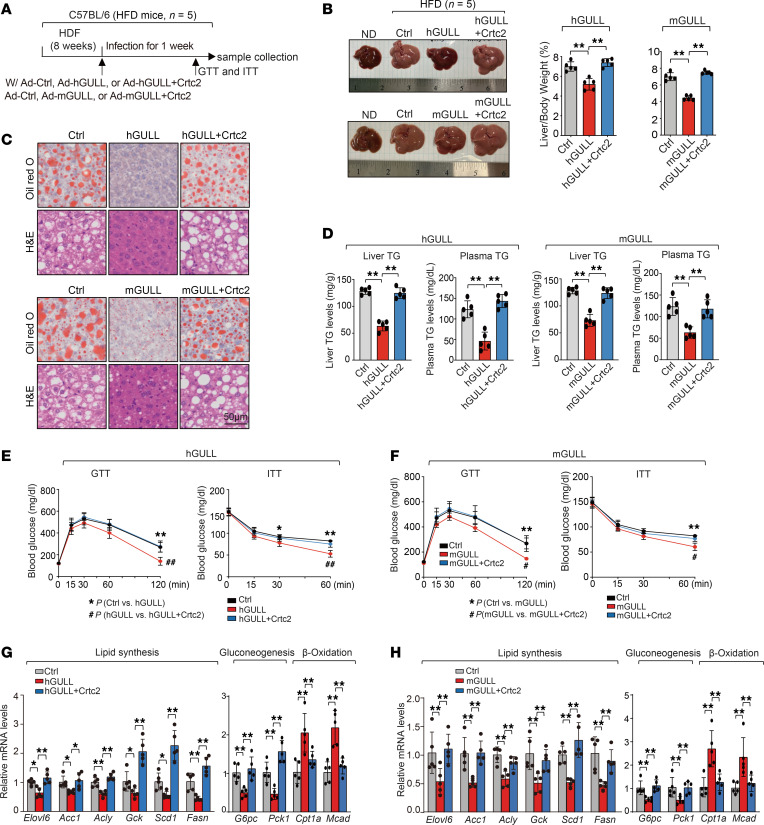
hGULL/mGULL ameliorate obesity-induced metabolic abnormalities in a CRTC2-dependent manner. (**A**) Graphical representation of obesity mouse model to explore the phenotype and molecular mechanisms of hGULL/mGULL and CRTC2. Mice were injected with 5 groups of adenoviruses: the Ad-Ctrl group (pAdV5), hGULL overexpression group, hGULL+Crtc2 overexpression group, mGULL overexpression group, or mGULL+Crtc2 overexpression group. (**B**–**D**) Representative images of liver and liver/body weight ratio analysis (**B**), Oil Red O and H&E staining (**C**), and plasma/liver TG level analysis (**D**) in Ad-Ctrl group, hGULL or mGULL overexpression group, and hGULL+Crtc2 or mGULL+Crtc2 overexpression group. **P < 0.01; data shown as mean ± SD, 1-way ANOVA. Scale bar: 50 μm. (**E** and **F**) GTT and ITT were determined in the Ad-Ctrl group, hGULL or mGULL overexpression group, and hGULL+Crtc2 or mGULL+Crtc2 overexpression group. **P < 0.01, *P < 0.05, ^##^*P* < 0.01, ^#^*P* < 0.05; data shown as mean ± SD, 1-way ANOVA. (**G** and **H**) The mRNA levels of lipid synthesis genes (*Elovl6*, *Acc1*, *Acly*, *Gck*, *Scd1*, and *Fasn*), gluconeogenesis genes (*G6pc* and *Pck1*), and β-oxidation genes (*Cpt1a* and *Mcad*) were quantified in Ad-Ctrl group, hGULL or mGULL overexpression group, and hGULL+Crtc2 or mGULL+Crtc2 overexpression group using qPCR. *P < 0.05, **P < 0.01; data shown as mean ± SD, 1-way ANOVA.

The robust lipid- and glucose-lowering effects of hGULL/mGULL suggest that they might be able to improve defective lipid and glucose metabolism in obese mice. To investigate this, we overexpressed hGULL or mGULL in the livers of mice with diet-induced obesity (Figure 4A) and found that both resulted in significant decrease in liver size and the liver/body weight ratio (Figure 4B). Overexpression of hGULL or mGULL also reduced neutral lipid levels in liver tissue and decreased liver and plasma TG levels (Figure 4, C and D). Moreover, GTT and ITT showed significantly improved glucose disposal rates in hGULL- or mGULL-overexpressing mice (Figure 4, E and F). Consistent with these results, the expression levels of lipogenic genes and gluconeogenesis genes were all decreased by hGULL/mGULL overexpression, while β-oxidation gene expression showed the opposite pattern (Figure 4, G and H). Remarkably, all these beneficial effects mediated by hGULL/mGULL overexpression were significantly attenuated by Crtc2 overexpression (Figure 4, B–H). These results not only reinforce our earlier findings that hGULL/mGULL are FCLs that modulate the function of CRTC2 but also indicate potential therapeutic benefits against obesity-induced metabolic abnormalities.

### Long-term expression of human and mouse GULLs by adeno-associated virus robustly ameliorates obesity-induced metabolic abnormalities.

**Figure 5:**
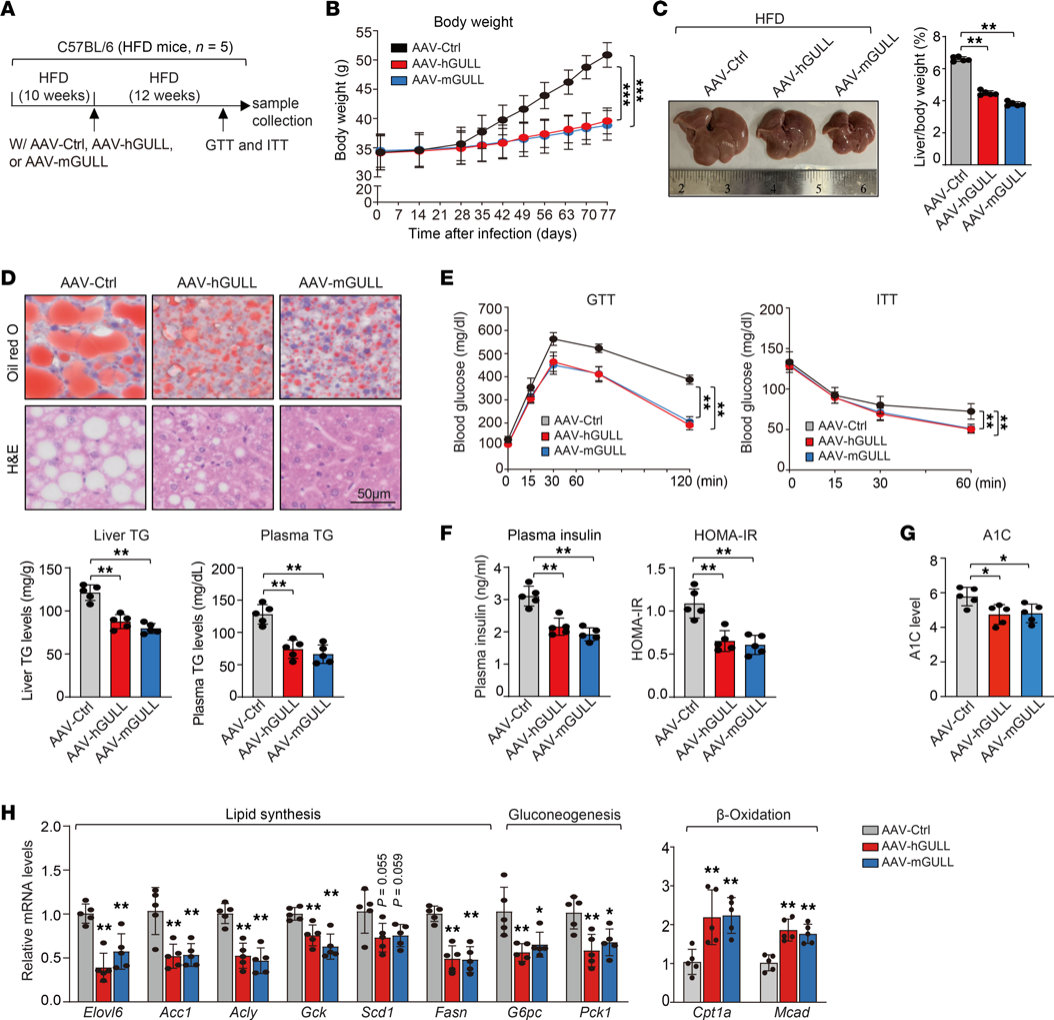
AAV-mediated hGULL/mGULL overexpression improves metabolic health of obese mice. (**A**) Graphical representation of AAV-obesity mouse model to explore the phenotype of hGULL/mGULL. Mice were injected with 3 groups of AAVs: AAV-Ctrl, AAV-hGULL, and AAV-mGULL. (**B**) Statistical analysis of body weight in the AAV-Ctrl, AAV-hGULL, and AAV-mGULL groups. ***P < 0.001. (**C** and **D**) Representative images of liver and liver/body weight ratio analysis (**C**), Oil Red O and H&E staining, and plasma/liver TG level analysis (**D**) in the AAV-Ctrl, AAV-hGULL, and AAV-mGULL groups. **P < 0.01; data shown as mean ± SD, 1-way ANOVA. Scale bar: 50 μm. (**E**) GTT and ITT were determined in the AAV-Ctrl, AAV-hGULL, and AAV-mGULL groups. **P < 0.01; data shown as mean ± SD, 1-way ANOVA. (**F** and **G**) Plasma insulin level, HOMA-IR (**F**), and hemoglobin A_1c_ level (**G**) were analyzed in the AAV-Ctrl, AAV-hGULL, and AAV-mGULL groups. *P < 0.05, **P < 0.01; data shown as mean ± SD, 1-way ANOVA. (**H**) The mRNA levels of lipid synthesis, gluconeogenesis, and β-oxidation genes were quantified in the AAV-Ctrl, AAV-hGULL, and AAV-mGULL groups using qPCR. *P < 0.05, **P < 0.01; data shown as mean ± SD, 1-way ANOVA.

Adenovirus-mediated gene expression in the liver is typically short-lived and often triggers strong inflammatory responses (22). To examine the long-term effects of hGULL/mGULL while minimizing potential immune response effects, we used adeno-associated virus (AAV) to express hGULL/mGULL in mice with high-fat diet–induced obesity (Figure 5A). After 3 months, mice receiving hGULL or mGULL AAVs (AAV-hGULL/mGULL) exhibited a significant decrease in body weight compared with the control group (Figure 5B). The liver size and liver/body weight ratio also decreased in the AAV-hGULL/mGULL group (Figure 5C). Consistent with the adenovirus-mediated overexpression results, neutral lipid levels in liver tissue and TG levels in liver and plasma were significantly decreased in the AAV-hGULL/mGULL group (Figure 5D). Furthermore, glucose disposal in GTT and ITT were significantly improved in the AAV-hGULL/mGULL group (Figure 5E). Fasting insulin levels in plasma and the homeostatic model assessment of insulin resistance (HOMA-IR) index were also decreased in the AAV-hGULL/mGULL group (Figure 5F). To assess the long-term effects of hGULL/mGULL on blood glucose levels, we measured hemoglobin A1c levels, which were significantly decreased in the AAV-hGULL/mGULL group (Figure 5G). Additionally, the expression levels of lipogenic genes and gluconeogenesis genes were significantly decreased, whereas those of β-oxidation genes were significantly increased, in AAV-hGULL/mGULL mice (Figure 5H). Collectively, these results demonstrate that long-term hGULL or mGULL expression substantially ameliorates fatty liver and improves insulin sensitivity and glucose metabolism in obese mice.

### Functional motifs of GULLs, GULFs, strongly improve metabolic health in obese mice.

**Figure 6:**
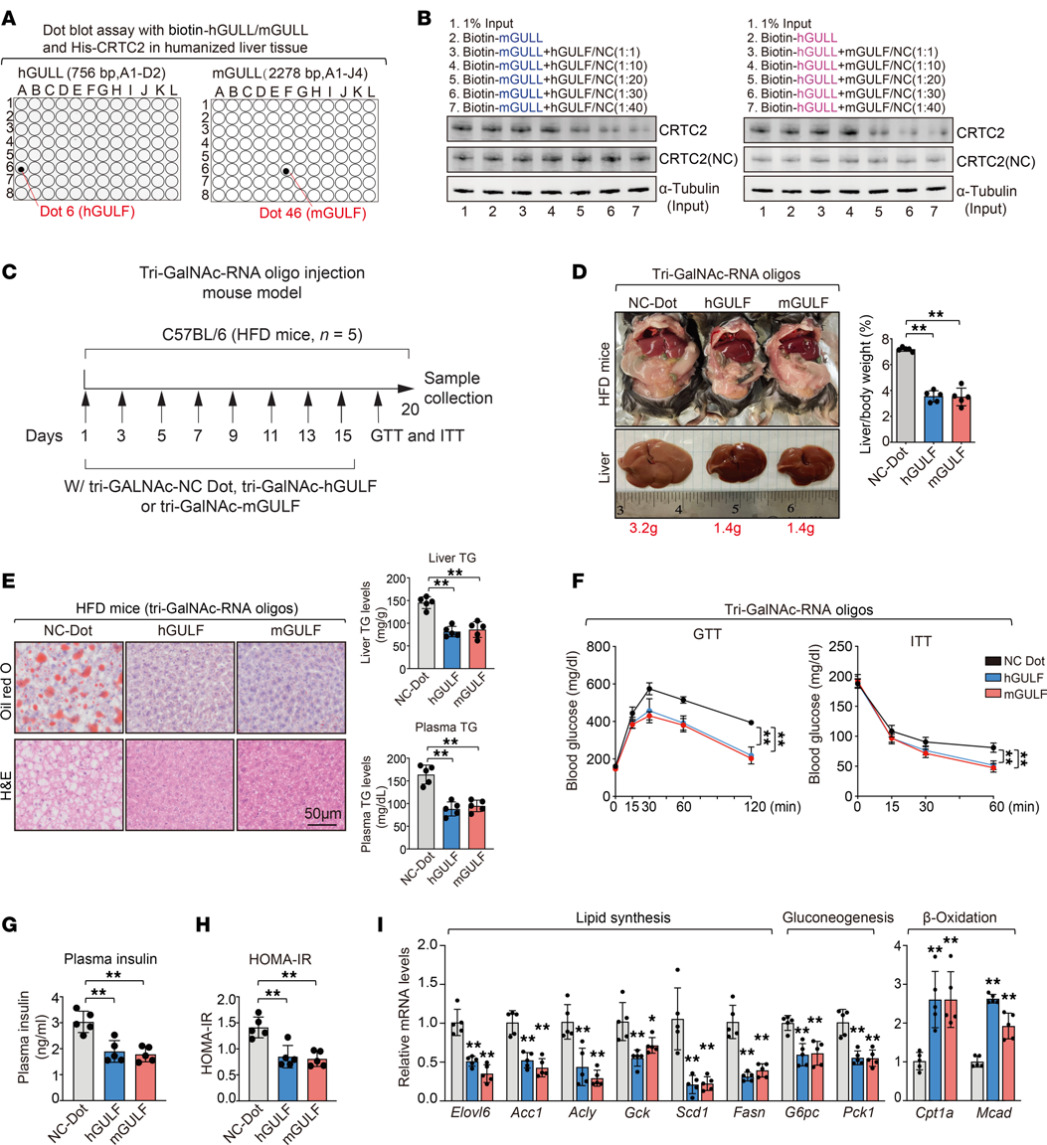
hGULF/mGULF improve metabolic health of obese mice. (**A**) RNA dot blot assays were performed to identify the core binding motif between CRTC2 and hGULL/mGULL. (**B**) hGULL/mGULF competition assay coupled with RNA pull-down and visualized by immunoblot assay. Non-biotin-labeled hGULF or mGULF with different doses was used to compete with biotin-labeled hGULL or mGULL (mole ratio of hGULL/mGULL to hGULF/mGULF: 1:1, 1:10, 1:20, 1:30, and 1:40) for binding to the CRTC2. (**C**) Graphical illustration shows the treatment schedules of tri-GalNAc–tagged hGULF/mGULF mimics and NC dot oligos in the high-fat diet (HFD) mouse model. Mice were injected with tri-GalNAc-NC oligos, tri-GalNAc-hGULF oligos, and tri-GalNAc-mGULF oligos every 2 days for 2 weeks. (**D** and **E**) Representative images of the liver and liver/body weight ratio analysis (**D**), Oil Red O and H&E staining, and plasma/liver TG level analysis (**E**) in tri-GalNAc-NC, tri-GalNAc-hGULF, and tri-GalNAc-mGULF groups. **P < 0.01; data shown as mean ± SD, 1-way ANOVA. Scale bar: 50 μm. (**F**) GTT and ITT were determined in the tri-GalNAc-NC, tri-GalNAc-hGULF, and tri-GalNAc-mGULF groups. **P < 0.01; data shown as mean ± SD, 1-way ANOVA. (**G** and **H**) Plasma insulin level (**G**) and HOMA-IR (**H**) were determined in tri-GalNAc-NC, tri-GalNAc-hGULF, and tri-GalNAc-mGULF groups. **P < 0.01; data shown as mean ± SD, 1-way ANOVA. (**I**) The mRNA levels of lipid synthesis, gluconeogenesis, and β-oxidation genes were quantified in the tri-GalNAc-NC, tri-GalNAc-hGULF, and tri-GalNAc-mGULF groups with qPCR. *P < 0.05, **P < 0.01; data shown as mean ± SD, 1-way ANOVA.

The challenge of developing effective lncRNA drugs is partly rooted in the sequence divergence of lncRNAs among species, which impedes the identification of key functional elements on human lncRNAs that can serve as direct targets of therapy development. As hGULL and mGULL carry out similar functions via their specific binding to CRTC2 in humans and mice, their respective CRTC2 binding motifs might be essential to their function and can serve as potential therapeutic targets. To identify the core motifs of hGULL/mGULL essential for the CRTC2 interactions, we conducted an in vitro biotin-RNA-protein binding coupled with dot blot assay to scan hGULL/mGULL for their specific binding sites to CRTC2. We identified that CRTC2 interacted with hGULL at 136–166 nt (dot 6, A6: 5′-UGUGGCAUGAAGAGGUCAGGCCAUUCCAGC-3′) and with mGULL at 1,349–1,379 nt (dot 46, F6: 5′-UUGCCAAACCUUUCUUGGUGAGCUGGAUGC-3′) (Figure 6A). We named these motifs GULFs (GULLs’ functional motifs). To determine the significance of human and mouse GULFs to the interaction between GULL and CRTC2, we performed competitive binding assays and found that mGULF was able to effectively compete with full-length hGULL in its binding to CRTC2 in a dose-dependent manner (Figure 6B). Similarly, hGULF was also able to compete with full-length mGULL (Figure 6B). These findings indicate that hGULF or mGULF specifically mediates the interaction between hGULL/mGULL and CRTC2. Furthermore, the predicted secondary structures of hGULF and mGULF are similar, providing a possible reason for their interaction with CRTC2 (Supplemental Figure 2C).

The robust binding activities of hGULF/mGULF toward CRTC2 suggest that they might play an important role in the regulation of CRTC2 function by hGULL/mGULL and they might have similar function to their full-length counterparts in lipid and glucose metabolism. To investigate this intriguing possibility, we used several systems to examine the impact of hGULF/mGULF on lipid and glucose metabolism. First, we used adenovirus to express hGULF or mGULF in the livers of mice with high-fat diet–induced obesity (Supplemental Figure 3A) and found that they exhibited all the same beneficial effects as their full-length counterparts. For example, hGULF/mGULF-expressing mice exhibited reduced liver size and liver/body weight ratio (Supplemental Figure 3B). The neutral lipid levels in liver tissue and TG levels in the liver or plasma were also significantly decreased in these mice (Supplemental Figure 3C). Furthermore, glucose disposal in GTT and ITT was significantly improved in hGULF/mGULF-expressing mice (Supplemental Figure 3D). Finally, the expression levels of lipogenic and glucogenic genes were reduced and insulin sensitivity was increased upon h/mGULF expression (Supplemental Figure 3, E and F).

Next, we investigated whether standalone GULFs also affect lipid and glucose metabolism. We synthesized hGULF/mGULF RNA oligonucleotides (oligos) and added tri-*N*-acetylgalactosamine (tri-GalNAc) tag to facilitate their delivery to hepatocytes. We then transfected the hGULF or mGULF oligos into mouse primary hepatocytes and found that they significantly reduced the expression levels of lipogenic and gluconeogenic genes induced by palmitic acid treatment (Supplemental Figure 4A). Consistent with the reduced expression of gluconeogenic genes, hGULF oligos also reduced glucose production in mouse primary hepatocytes (Supplemental Figure 4B). Intriguingly, the reduced glucose production by hGULF oligos was reversed by mGULL knockdown (Supplemental Figure 4B), supporting that hGULF and mGULL have a similar function in modulating gluconeogenesis in hepatocytes. Moreover, hGULF oligos also increased both basal and insulin-stimulated phosphorylation of AKT and GSK3β, and this effect was also reversed by mGULL knockdown (Supplemental Figure 4C).

Finally, to understand whether these standalone motifs have lipid- and glucose-lowering effects in vivo, we applied tri-GalNAc–tagged hGULF/mGULF oligos into mice with diet-induced obesity via intravenous and subcutaneous injection (23) (Figure 6C). Tri-GalNAc-h/mGULFs exhibited liver-specific distribution in mice, with levels detectable between 6 and 48 hours after injection and peaking at 12 hours (Supplemental Figure 5, A and B).

**Figure 7:**
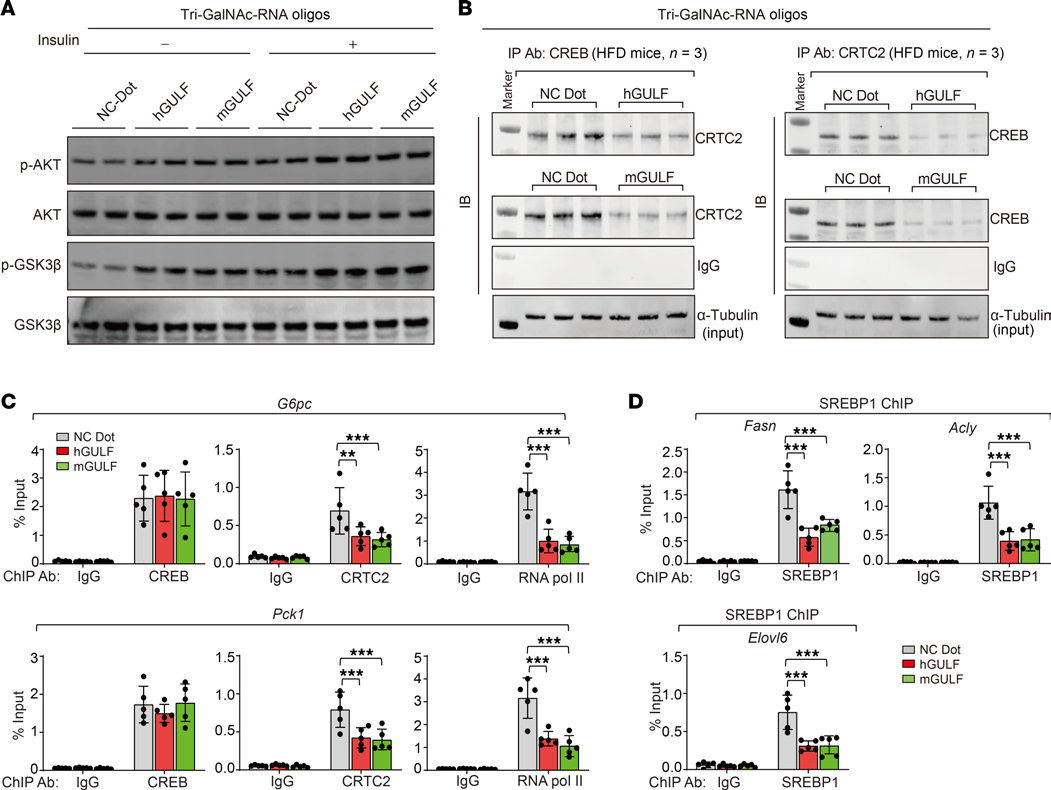
hGULF and mGULF regulate the function of CREB and SREBP1. (**A**) Immunoblotting of p-AKT, AKT, p-GSK3β, and GSK3β in the tri-GalNAc-NC, tri-GalNAc-hGULF, and tri-GalNAc-mGULF groups with or without insulin treatment. (**B**) Coimmunoprecipitation to detect the association between CRTC2 and CREB after tri-GalNAc-hGULF/mGULF injection. Mice were treated with 6 hours of fasting before sacrifice. (**C**) ChIP-qPCR detection of occupancy of CREB or CRTC2 and RNA polymerase II level on *G6pc* and *Pck1* promoter using the liver tissues from tri-GalNAc-NC, tri-GalNAc-hGULF, and tri-GalNAc-mGULF groups. IgG was used as negative control. **P < 0.01, ***P < 0.001; data shown as mean ± SD, 2-way ANOVA. (**D**) ChIP-qPCR detected SREBP1 occupancy on *Fasn*, *Acly*, and *Elovl6* promoters with liver tissues from mice of the tri-GalNAc-NC, tri-GalNAc-hGULF, and tri-GalNAc-mGULF groups. IgG was used as negative control. ***P < 0.001; data shown as mean ± SD, 2-way ANOVA.

Body weight was modestly decreased in the tri-GalNAc-hGULF or tri-GalNAc-mGULF group, though food intake was not changed (data not shown). Tri-GalNAc-hGULF/mGULF injection resulted in significant decreases in liver size and liver/body weight ratio (Figure 6D) as well as neutral lipid levels in liver tissue and TG levels in liver and plasma (Figure 6E). Glucose metabolism was also significantly improved in mice receiving tri-GalNAc-hGULF/mGULF injection as shown by increased glucose disposal rates in GTT and ITT (Figure 6F) and decreased fasting insulin levels and HOMA-IR index (Figure 6, G and H). Consistently, the expression levels of lipogenic and gluconeogenic genes in mouse livers were also significantly decreased by hGULF/mGULF oligos, whereas the expression levels of β-oxidation genes were increased (Figure 6I). Furthermore, hGULF/mGULF oligos also increased basal and insulin-stimulated p-AKT and p-GSK3β in mouse livers (Figure 7A).

Finally, we attempted to understand the mechanisms of action of tri-GalNAc–tagged hGULF/mGULF oligos in a pathophysiological context. We found that in the livers of obese mice receiving tri-GalNAc–tagged hGULF or mGULF oligos, the interaction between CREB and CRTC2 was significantly reduced (Figure 7B), supporting that hGULF and mGULF, like their full-length counterparts, can also disrupt CREB-CRTC2 interaction. Consistently, a ChIP assay showed that the occupancy of CRTC2 and RNA polymerase II at the promoter region of gluconeogenic genes (*G6pc* and *Pck1*) was significantly decreased in the tri-GalNAc-hGULF/mGULF injection group (Figure 7C), which could explain the reduced expression levels of these genes. Moreover, the occupancy of SREBP1 at the promoter region of lipid synthesis genes (*Fasn*, *Acly*, and *Elovl6*) was decreased in the tri-GalNAc-hGULF/mGULF injection group, which showed the same effects as the full-length hGULL/mGULL in vivo (Figure 7D).

Taken together, these results support that hGULF/mGULF, as a key functional motif that binds to CRTC2, can perform a similar biological function to full-length hGULL/mGULL in vivo and in vitro. Standalone hGULF/mGULF oligos can effectively improve insulin sensitivity and decrease glucose or lipid levels in obese mice, supporting their potential as potent glucose- and lipid-lowering therapeutics.

## Discussion

Although growing evidence has connected lncRNAs to the pathogenesis of metabolic diseases, no drugs targeting human lncRNAs have been approved by the FDA. This gap is partly due to the limited conservation of human lncRNAs, which makes it uniquely challenging to identify those playing critical roles in metabolic physiology, experimentally define their functions, and pinpoint specific sites on these lncRNAs as viable drug development targets.

In this work, we developed a framework addressing these challenges and identified a functional human lncRNA motif with significant therapeutic potential. First, we combined eQTL mapping and functional conservation analyses to select human lncRNAs genetically associated with metabolic diseases and with strong functional potential. We identified over 287 human lncRNAs whose expression levels are affected by metabolic disease–associated SNPs in the liver. Given that most of these lncRNAs are nonconserved, it would be difficult to ascertain whether any are functional. Our subsequent functional conservation analysis quickly reduced this number to 7, all of which have potential functionally conserved equivalents in mice. Growing evidence supports that functionally conserved lncRNAs are a prevalent phenomenon (8, 9), raising the possibility that some or all of these 7 lncRNAs might have conserved physiological significance. Second, we carried out extensive in vivo functional analyses to verify that 1 pair of selected lncRNAs, hGULL/mGULL, are legitimate FCLs and play important roles in maintaining metabolic homeostasis. The limited conservation of human lncRNAs makes it challenging to experimentally define their in vivo function, but we leveraged the concept of FCL to address this hurdle. We found that mGULL exhibited strong glucose- and lipid-lowering effects and hGULL fully mirrored its function. Both bind to CRTC2 and inhibit its function toward CREB and SREBP1, providing a clear mechanistic basis for their function. Importantly, they are effective in restoring proper glucose and lipid metabolism in obese mice, crucial evidence that warrants further investigation of hGULL as a drug target. Third, we used an innovative approach to identify the specific site on hGULL as a viable drug target and test its therapeutic potential using an FDA-approved drug delivery platform. The size of lncRNAs and challenges in resolving their structures make it difficult to pinpoint their functional domains or motifs. We addressed this by directly mapping the binding sites of hGULL/mGULL to their protein-binding partner, CRTC2, which we named GULF. Intriguingly, GULFs on both hGULL and mGULL have glucose- and lipid-lowering effects similar to those of their full-length counterparts. More critically, synthesized GULFs, similar to FDA-approved siRNA drugs in terms of size and modification, exhibit comparable effects. As GULF is part of naturally produced human lncRNAs, its anticipated toxicity and side effects were minimal, giving it a clear path for further clinical investigation.

CRTC2 is a key metabolic regulator, and GULL represents an important lncRNA-based mechanism modulating CRTC2 function. We have shown that mGULL regulates the activity of 2 critical downstream effectors of CRTC2, namely CREB and SREBP. In addition to these roles, CRTC2 influences lipid metabolism by regulating autophagy — particularly lipophagy — which is essential for maintaining lipid homeostasis (15, 24, 25). Here, we also investigated whether mGULL regulates lipophagy. We found that Crtc2 overexpression significantly reduced lipophagy, and this effect was reversed by either mGULL or hGULL overexpression (Supplemental Figure 6A). Consistently, the mRNA levels of key autophagy-related genes — including *Atg5*, *Atg7*, *Ulk1*, *Tfeb*, and *Atgl* — were similarly restored (Supplemental Figure 6B). These findings demonstrate that Crtc2 overexpression suppresses autophagy, and that this suppression is counteracted by h/mGULL, supporting a role for GULL in regulating this important facet of CRTC2 activity. Furthermore, mGULL appears to act as a tissue-specific regulator of CRTC2 function. CRTC2 is known to play a pivotal role in controlling body weight, global fatty acid metabolism, and glucose homeostasis across multiple tissues, including the liver, skeletal muscle, and adipose tissue (12, 26, 27). We found that h/mGULL showed relatively higher RNA levels in the liver and skeletal muscle but not in the heart or adipose tissues (Supplemental Figure 7, A and B). However, under both physiological and pathological conditions, skeletal muscle exhibited no significant changes in h/mGULL expression (Supplemental Figure 7, C–E). These findings highlight the liver as the primary site of GULL-mediated regulation of systemic metabolism.

The identification of hGULL and hGULF represents a significant advancement toward developing an lncRNA-based drug for metabolic disorders. As discussed, we have provided multiple lines of evidence supporting the crucial role of hGULL/mGULL in glucose and lipid metabolism. This includes their genetic association with metabolic diseases, substantial functional impact on lipid and glucose metabolism, and specific interaction with a known metabolic regulator. Targeting hGULL by delivering its functional motif, GULF, offers several advantages over modulating protein-coding genes downstream or upstream of hGULL. For instance, dominant-negative CREB in the heart can cause cardiac hypertrophy (28), a risk that would be mitigated by specific interaction of hGULF with CRTC2 in the liver. Furthermore, GULL expression is strongly induced by FXR activation, suggesting that FXR agonists might have beneficial effects similar to those of GULF. Indeed, obeticholic acid (OCA; INT-747), a bile acid–derived FXR agonist, is currently in phase III clinical trials for metabolic dysfunction–associated steatohepatitis treatment (29). However, OCA has a notable adverse effect of increasing LDL-cholesterol levels (30). Interestingly, we found that LDL-cholesterol levels in mice receiving hGULF and mGULF oligos were reduced, presenting a clear contrast to OCA (Supplemental Figure 8, A and B).

In summary, we have leveraged the concept of functionally conserved lncRNAs to develop a framework for selecting human lncRNAs as viable drug candidates. Our approach identified hGULL as a human lncRNA genetically associated with metabolic disorders and with a potential functional counterpart in mice. We performed extensive functional analyses in primary hepatocytes and regular and obese mice, verifying that hGULL is a legitimate FCL with significant impacts on glucose and lipid metabolism. Finally, we demonstrated that the CRTC2-binding site of hGULL, hGULF, is a functionally conserved human lncRNA motif with strong potential as a glucose- and lipid-lowering therapeutic.

## Methods

Additional details of the methods are provided in Supplemental Methods.

### Sex as a biological variable

All mice in our study were male. Compared with female mice, males can minimize the impact of estrogen fluctuations on metabolism and are more sensitive to metabolic disorders induced by a high-fat diet (31, 32). While these findings are directly applicable to males, further studies are needed to confirm their relevance in females, as sex differences may influence metabolic responses.

### GWAS-eQTL analysis

The GWAS data were obtained from the GWAS Catalog (https://www.ebi.ac.uk/gwas/home). Significant SNPs associated with metabolic disorders were identified as loci with a *P* value less than 10^–8^ and linked to metabolic traits. The eQTL analysis was conducted as previously described, following the GTEx consortium protocols (http://github.com/broadinstitute/gtex-pipeline) (5). In brief, liver eQTL discovery was performed using GTEx v7 RNA-Seq data and whole-genome sequencing (WGS) genotyping data (*n* = 118 samples). The GTEx RNA-Seq data were quantified using our custom liver-specific annotation, generated as previously described (10), and normalized using trimmed mean of M-values methods (33). QTL analysis was carried out using FastQTL with the default settings (34). The analysis included the top 3 genotyping principal components, 15 probabilistic estimation of expression residuals factors, sex, and genotyping platform as covariates (35). All lncRNA genes with an eQTL false discovery rate (FDR) less than 0.05 were selected for overlapping with the functionally conserved lncRNA database.

### Adenovirus generation and in vivo adenovirus injection

Full-length hGULL, mGULL, and Crtc2 were cloned into pDONR221 vector (catalog 12536017, Thermo Fisher Scientific) and then used to construct pAdv5 adenovirus vectors using the Gateway LR reaction system (catalog 11791020, Thermo Fisher Scientific). For shRNAs and h/mGULF, the oligos with the hairpin structure were cloned into the pAd/BLOCK-iT-DEST RNAi Gateway Vector (catalog V49220, Thermo Fisher Scientific) according to the protocols. Adenoviral plasmids were then digested with PacI enzyme (catalog R0547L, New England Biolabs), purified (MinElute Reaction Cleanup Kit, catalog 28206, QIAGEN), and transfected into HEK293A cells with Lipofectamine 2000 Transfection Reagent (catalog 11668027, Thermo Fisher Scientific) in a 6-well plate for initial crude virus generation. At 48 hours later, cells were passaged to 10 cm dishes, and crude virus was collected after 5–10 days. Crude virus was then purified through 50% CsCl ultracentrifugation (137,000*g* overnight). The purified viruses were then desalted using PD10 columns (catalog 17085101, Cytiva) and titered using the Adeno-X Rapid Titer Kit (catalog 632250).

Adenoviruses were delivered via retro-orbital injection under anesthesia and aseptic conditions. Mice were anesthetized with intraperitoneal (i.p.) administration of ketamine (80–120 mg/kg; catalog NDC 13985-584-10, NIH Pharmacy) and xylazine (5–25 mg/kg; catalog NDC 59399-110-20, NIH Pharmacy). Using a 30G needle (catalog 305106, BD), 0.1 mL of virus particles suspended in sterile PBS were carefully injected into the orbital venous plexus. Then, mice were placed in cages without bedding on a warming pad and closely monitored until they fully recovered from anesthesia, after which they were returned to their home cages.

### Short-read RNA-Seq analysis and Gene Ontology pathway enrichment analyses

Total RNA was isolated with the MagMAX-96 Total RNA Isolation Kit (catalog AM1830, Thermo Fisher Scientific) before sequencing. Strand-specific sequencing libraries were constructed using the Illumina TruSeq RNA sample prep kit, and sequencing was conducted at the National Heart, Lung, and Blood Institute DNA Sequencing and Genomics Core. Analysis of mouse short-read RNA-Seq data was carried out as previously described (36). Briefly, the FASTQ read files were initially trimmed and cleaned using fastp/0.23.2 and assessed for quality with FastQC/0.11.8 (https://www.bioinformatics.babraham.ac.uk/projects/fastqc). The reads were then aligned using HISAT2/2.2.1.0 (https://daehwankimlab.github.io/hisat2) with an index created from the GRCm38.p6 genome. Aligned reads were counted with GENCODE VM24 (https://www.gencodegenes.org/mouse/release_M24.html) as the reference. Differential gene expression analysis was conducted in the same manner as the humanized mouse RNA-Seq analysis. A log_2_(fold change) cutoff greater than 0.5 and a *P* value lower than 0.05 were used to identify differentially expressed genes. Genes commonly altered in the mGULL knockdown and hGULL rescue groups were subjected to Gene Ontology pathway enrichment analysis, which was performed and visualized using the R package clusterProfiler/3.18.0 with default settings (https://github.com/YuLab-SMU/clusterProfiler). The heatmap was generated through the heatmap package on R Studio.

### Animal assays

#### Adenovirus injection in C57BL/6 wild-type mice.

Male 7-week-old C57BL/6 mice were intravitreally injected with adenovirus expressing control, shmGULL, or shmGULL+hGULL (control group: Lac+pAdV5; shmGULL group: sh-mGULL+pAdV5). For each group, total active viral particles were 5 × 10^8^ plaque-forming units (PFU). Mice were sacrificed 7 days after injection (see Figure 1C). Seven-week-old C57BL/6 male mice were injected with Ad-Control, Ad-Crtc2, Ad-Crtc2+hGULL, or Ad-Crtc2+mGULL (control group: Lac+pAdV5; Crtc2 group: Lac+Crtc2) once a week for 3 weeks with continued feeding of a regular diet to mimic obesity condition (see Figure 2C). For each group, total active viral particles were 5 × 10^8^ PFU.

#### Virus injection in C57BL/6J mice with diet-induced obesity.

Male 8- to 10-week-old C57BL/6J mice with diet-induced obesity (DIO mice) (The Jackson Laboratory; high-fat diet: catalog D12492, Research Diets Inc.) were injected with Ad-Control, Ad-hGULL, or Ad-hGULL+Crtc2, or Ad-Control, Ad-mGULL, or Ad-mGULL+Crtc2, for 7 days before sacrifice (see Figure 4A), and injected with AAV-Control, AAV-hGULL, or Ad-mGULL (for each group, total active viral particles were 5 × 10^9^ PFU) for 12 weeks before sacrifice (see Figure 5A). All AAVs were purchased from AAVnerGene with pAAV.TBG.PI.Null.bGH (catalog 105536, Addgene) as the backbone. For functional motif of hGULF or mGULF in dietary obese mice, DIO C57BL/6J 10-week-old males were injected with tri-GalNAc-hGULF RNA oligos, tri-GalNAc-mGULF RNA oligos, or tri-GalNAc-NC RNA oligos every 2 days for 2 weeks (for each tri-GalNAc RNA, 0.01 mg/g per mouse; see Figure 6C) or injected with Ad-hGULF, Ad-mGULF, or Ad-NC (5 × 10^8^ PFU/mouse; see Supplemental Figure 3A) for 7 days before sacrifice.

#### Glucose tolerance test, insulin tolerance test, HOMA-IR, and hemoglobin A1c detection.

For glucose tolerance test, the mice were fasted overnight and injected i.p. with d-glucose (2 g/kg; catalog G7528, MilliporeSigma), and for insulin tolerance test, the mice were fasted for 5 hours and injected with insulin (1 U/kg; catalog 1342106, MilliporeSigma). Blood glucose levels were determined from tail veins using a portable Accu-Chek glucose meter (Roche) at 0, 15, 30, 60, and 120 minutes after injection. Plasma insulin levels were determined using the Ultra Sensitive Mouse Insulin ELISA Kit (catalog 90082, Crystal Chem). The HOMA-IR value was calculated using the formula HOMA-IR = fasting glucose × fasting insulin/405 (37). Hemoglobin A1c levels were determined using a Mouse Hemoglobin A1c Assay Kit (catalog 80310, Crystal Chem) in whole blood.

#### Mouse treatment for detection of insulin signaling pathway.

Mice were injected i.p. with insulin (0.25 U/kg) for 10 minutes, and p-AKT and p-GSK levels in liver extracts were detected by immunoblot assay. (A list of antibodies used in this study is shown in Supplemental Table 6.) Liver and plasma TG levels were measured using a Triglyceride Quantification Colorimetric/Fluorometric Kit (catalog MAK266-1KT, MilliporeSigma). Liver tissue was frozen in Tissue-Tek O.C.T. Compound (catalog 4583, Sakura Finetek) or fixed with 10% formalin (catalog HT501850, MilliporeSigma), sectioned, and stained with Oil Red O (catalog MEK194, MilliporeSigma) or H&E (catalog ab245880, Abcam). The staining intensities were quantified using ImageJ software (NIH).

#### Measurement of plasma HDL-cholesterol and LDL-cholesterol.

Plasma HDL- and LDL-cholesterol levels were quantified using enzymatic methods in accordance with the manufacturer’s protocols, using the Mouse HDL-Cholesterol Assay Kit (catalog 79990, Crystal Chem) and Mouse LDL-Cholesterol Assay Kit (catalog 79980, Crystal Chem), respectively.

### Glucose production assay

Human primary hepatocytes were transfected with hGULF RNA oligos and infected with Ad-shmGULL and after 24 hours were incubated overnight in serum, glucose, and phenol red–free DMEM containing 20 mM sodium lactate, 2 mM sodium pyruvate, 2 mM l-glutamine, and 15 mM HEPES. Fresh medium containing palmitic acid (300 μM; catalog P5585, MilliporeSigma) was added. After 24 hours, glucose concentrations in the medium were measured with a Glucose Assay Kit (catalog 11001-718, Wako Life Sciences). Reads were normalized to the total protein content determined from whole-cell lysates using the bicinchoninic acid protein assay kit (catalog 23225, Thermo Fisher Scientific).

### Synthesis of GalNAc-RNA mimics and in vivo injection

Small core functional motif RNA mimics (hGULF, mGULF, and NC dot) used in this study were synthesized by Bio-Synthesis Inc. All sequences are provided in Supplemental Table 5. GalNAc-RNA mimics (0.01 mg/g) were injected i.p. or intravenously into DIO C57BL/6J male mice every 2 days for 2 weeks (23). The livers were collected after the final injection, tissues were subjected to Oil Red O or H&E staining, and total RNA was isolated using the MagMAX RNA extraction kit (catalog AM1830, Thermo Fisher Scientific) per the manufacturer’s instructions.

### RNA dot blot and competition assay

One microgram biotinylated h/mGULL was incubated with 50 ng recombinant c-Myc–tagged CRTC2 proteins (catalog TP307354, OriGene Technologies) in RNA-protein binding buffer (50 mM Tris-HCl [pH 7.9], 10 mM β-mercaptoethanol, 10% glycerol, 5 mM MgCl_2_, 100 mM KCl, 0.1% NP-40, 10 mg/mL yeast transfer RNA, 40 U/μL RNase inhibitor) on ice for 1 hour. The RNA-protein mixture was then transferred into an ultraviolet cross-linker (catalog CL-3000, Analytik Jena) with 150 mJ/cm^2^ radiation for 10 minutes. The mixture was digested with RNase A for 30 minutes at 37°C and then incubated with anti–c-Myc magnetic beads for 1 hour. The bead mixture was washed 3 times with 1× TBS-T buffer (25 mM Tris, 0.15 M NaCl, 0.05% Tween 20) and then digested with proteinase K, releasing protected RNA fragments. RNA was then incubated with DNA probes (Supplemental Table 5) in ice-cold 10 mM NaOH and 1 mM EDTA buffer and dropped on nitrocellulose membranes (catalog 926-31090, LI-COR) using the Bio-Dot apparatus (catalog 170-6545, 170-6547, Bio-Rad) at 65°C for 2 hours. The membrane was washed with washing buffer at 37°C, 50°C, and 65°C for 20 minutes each. Dots were visualized by streptavidin/Alexa Fluor 800 signals. The DNA probes were all synthesized at Integrated DNA Technologies (IDT). For the RNA competition assay, biotin-labeled h/mGULL or h/mGULF was mixed with an equimolar amount of nonbiotin-labeled h/mGULL. The procedure was otherwise the same as RNA pull-down. Biotin-h/mGULF RNA oligos were synthesized by IDT.

### Statistics

Data were analyzed by 2-tailed Student’s *t* test or 1- or 2-way ANOVA with FDR test for single or multiple comparisons as appropriate using GraphPad Prism (v9.0.2) software, and *P* less than 0.05 was considered statistically significant.

### Study approval

All animal experiments were conducted in compliance with protocols approved by the National Heart, Lung, and Blood Institute Animal Care and Use Committee.

### Data availability

The raw RNA-Seq data for the rescue assay shown in Figure 1 were deposited in the NCBI’s Gene Expression Omnibus database (accession GSE272715). Values for data points in all figures are reported in the Supporting Data Values file. The authors declare that all relevant data of this study are available upon reasonable request.

## Author contributions

ZL, SS, CJ, PL, and HC designed the workflow. ZL, SS, CJ, and YM performed the experiments. CJ and HS performed bioinformatics analysis. ZL, SS, CJ, and HC wrote the manuscript. HC obtained funding and conceived and supervised the study. The order of the co–first authors is based on the contribution to the project.

## Supplementary Material

Supplemental data

Unedited blot and gel images

Supplemental table 1

Supplemental table 2

Supplemental table 3

Supplemental table 4

Supplemental table 5

Supplemental table 6

Supporting data values

## Version 1 09/16/2025

Electronic publication
