# Supplementary material for: The functionally conserved human lncRNA motif GULF lowers glucose and lipid levels in obese mice
Source: J Clin Invest. 2025 Sep 16;135(18):e186355. doi: 10.1172/JCI186355 (PMC12435843; doi:10.1172/JCI186355)
Supplement: Supplemental data [file jci-135-186355-s156.pdf]

Supplemental material

The functionally conserved human lncRNA motif GULF lowers glucose and lipid levels in obese mice

Zhe Li*, Sunmi Seok*, Chengfei Jiang*, Ping Li, Yonghe Ma, Hang Sun and Haiming Cao

Cardiovascular Branch
National Heart, Lung and Blood Institute
National Institutes of Health
Bethesda, MD 20892, USA

*equally contributed

Correspondence
Haiming Cao, Ph.D.
Cardiovascular Branch
National Heart, Lung and Blood Institute
National Institutes of Health
Bethesda, MD 20892, USA
Phone: 301-402-9032, Fax: 301-480-0360
Email: haiming.cao@nih.gov

Supplemental Methods

RNA isolation and quantitative reverse transcription polymerase chain reaction (RT-qPCR)

TRIzol reagent (Cat no. 15596026, Thermo Fisher Scientific) was used to extract total RNA both from tissues and cells in this study. The reverse transcription process was performed using SuperScript III reverse transcriptase kit (Cat no. 18080044, Thermo Fisher Scientific). qPCR was conducted using FastStart Universal SYBR Green Master kit (Cat no. 04913914001, Roche Life Science, Washington DC, USA), with GAPDH or β-actin as reference genes. All primers employed are listed in **Supplemental Table 5**.

Chromatin Immunoprecipitation (ChIP) assay

ChIP assays were conducted using the Enzymatic Chromatin IP Kit (Cat no. 9005, Cell Signaling Technology, Danvers, MA, USA) following the manufacturer's protocols. In brief, liver tissue was minced, washed twice in PBS, and then incubated with 1.5% formaldehyde for 20 min at room temperature (RT), followed by incubation with 125 mM Glycine for 5 min at RT. Samples were then sonicated 4 times for 10 seconds each time. Then, the chromatin sample was precleared, and chromatin was immunoprecipitated using 1-2 μg of target antibody or IgG. The immune complexes were collected by incubation with magnetic beads for 1 h, washed 3 times, and then eluted and incubated overnight at 65 °C to reverse the crosslinking. DNA was subsequently isolated for quantification by qPCR. The primer sequences used are in **Supplemental Table 5**.

In vitro Transcription/Translation assay

In vitro transcription and translation were conducted using the In Vitro Transcription/Translation kit (Cat no. L1170, Promega, Madison, WI, USA) to determine the protein-coding potential of hGULL or mGULL in vitro. Full-length h/mGULL with a UAA stop codon was cloned into a circular plasmid with a T7 promoter. The luciferase gene was used as a positive control. The coupled transcription/translation reactions were set up in a single tube with T7 quick master mix, methionine, plasmid DNA template, and Biotin-lysyl-tRNA, and incubated at 30°C for 60-90 minutes. The synthesized proteins were analyzed by SDS-PAGE and detected using the Transcend Non-Radioactive Translation Detection System (Cat no. L5070, Promega).

RNA Pull-Down and RNA Immunoprecipitation (RIP) assay

For RNA pulldown assay, biotin-labeled h/mGULL or antisense h/mGULL RNAs were transcribed by in vitro transcription using the Biotin RNA Labeling mix (Cat no. 11685597910, Roche) and T7 RNA polymerase (Cat no. 10881767001, Roche) at 37°C for 2-3 hours. 2 µg of biotinylated RNAs were incubated with 1 mg of protein extracts from humanized liver tissues and prewashed M-270 Streptavidin beads (Cat no. 65306, Thermo Fisher Scientific) at 4°C overnight. The beads were washed five times with ice-cold RNA pulldown buffer (5M NaCl, 1M Tris PH 7.4, 0.5M EDTA, and 0.5%Triton-X 100) and boiled with SDS loading buffer. The pulldown samples with beads were then sent for proteomic analysis at the National Cancer Institute's Protein Characterization Laboratory (PCL).

For the RNA immunoprecipitation (RIP) assay, Liver tissues were lysed in RNA immunoprecipitation lysis buffer (150 NaCl, 20mM Tris PH 7.4, 1M EDTA and 0.5% Triton-X100) containing protease inhibitors (Cat no. 78440, Thermo Fisher Scientific) and Recombinant Ribonuclease Inhibitor (Cat no. 10777019, Thermo Fisher Scientific) using a Dounce homogenizer (Cat no. D9938, Millipore Sigma) for 15-20 strokes. The lysate was then centrifuged at 12,000 rpm for 10 minutes; the supernatant was then collected. For each RIP, 5µg IgG or target antibody were first incubated with 40µl Protein A/G beads (Cat no. 88802, Thermo Fisher Scientific) in 300µl RIP buffer supplemented with 0.1 mg/ml BSA (Cat no. A1595, Millipore Sigma), 0.2 mg/ml Heparin (Cat no. 1235831, Millipore Sigma) and 0.2mg/ml tRNA (Cat no. AM7119, Thermo Fisher Scientific) for 1 hour. Samples were then washed once with RIP buffer, the antibody coupled beads immunoprecipitated with 5mg liver tissue supernatant in 500µl RIP buffer at 4°C with gentle rotation overnight. The beads were washed briefly five times with RIP buffer, following proteinase K digestion, and total RNA was isolated with the TRIZOL method and purified with the columns (Cat no. R1014, ZYMO Research, Irvine, CA, USA). The RNAs were then resuspended in RNase-free water and qRT-PCR was performed as previously described.

Immunoblot and Co-IP

Liver tissues were snap-frozen in liquid nitrogen immediately upon harvesting. Frozen samples were lysed in RIPA lysis buffer (Cat no. 89900, Thermo Fisher Scientific) with freshly added

protease inhibitor cocktail and phenylmethylsulfonyl fluoride (PMSF, Cat no.36978, Thermo Fisher Scientific) using the Tissue Lyser III (Cat no. 9003240, Qiagen, Germantown, MD, USA) to extract proteins. Total protein concentration was quantified with the kit (Cat no.23225, Thermo Fisher Scientific), and 10–30μg total protein was run on a Bis-Tris polyacrylamide gel (Cat no. NP0322BOX, Thermo Fisher Scientific). Then, proteins were electro-transferred onto PVDF membranes with a transfer pack (Cat no. 1704158, Bio-Rad Laboratories, Hercules, CA, USA). Membranes were then incubated with blocking buffer (Cat no. 37515, Thermo Fisher Scientific) for 10 mins at room temperature, followed by incubation with primary antibodies overnight at 4°C. Membranes were then washed three times with PBST buffer and incubated at room temperature for 1 hour with fluorescence-conjugated secondary antibodies (Cat no. 926-54010, 926-54020, LI-COR Biosciences, Lincoln, NE, USA). The signals were visualized with the LI-COR Odyssey imaging system and analyzed by ImageJ (National Institutes of Health).

For Co-IP, the same procedures with IB in liver tissue protein preparation. The lysates were incubated with target proteins and IgG antibodies at room temperature for 1 hour and the washed protein A/G beads continued incubated at 4°C overnight. Following the wash steps with RIPA buffer, the IP proteins were subjected to IB for further analysis.

Reagents and Resource

Primary human hepatocytes (Cat no, HUM181001B) were purchased from Lonza Bioscience (Rockville, Maryland, USA) and C57BL/6 Mouse primary hepatocytes (Cat no. C57-6224F) were purchased from Cell biologics company (Chicago, Illinois, USA). Human tissue cDNA panel (Cat. no. HD-010) and mouse tissue cDNA panel (Cat. no. MD-010) were purchased from Zyagen company (San Diego, CA, USA). Human male adipose tissue cDNA purchased from BioChain Institute Inc. (Cat no. C123400310, Newark, CA, USA). C57BL/6 wide-type mice and DIO C57BL/6 background High-fat diet mice were obtained from the Jackson Laboratory (Bar Harbor, ME, USA). Details of Antibodies used in this study are shown in **Supplemental Table 6**.

Supplemental Figures and Figure legends

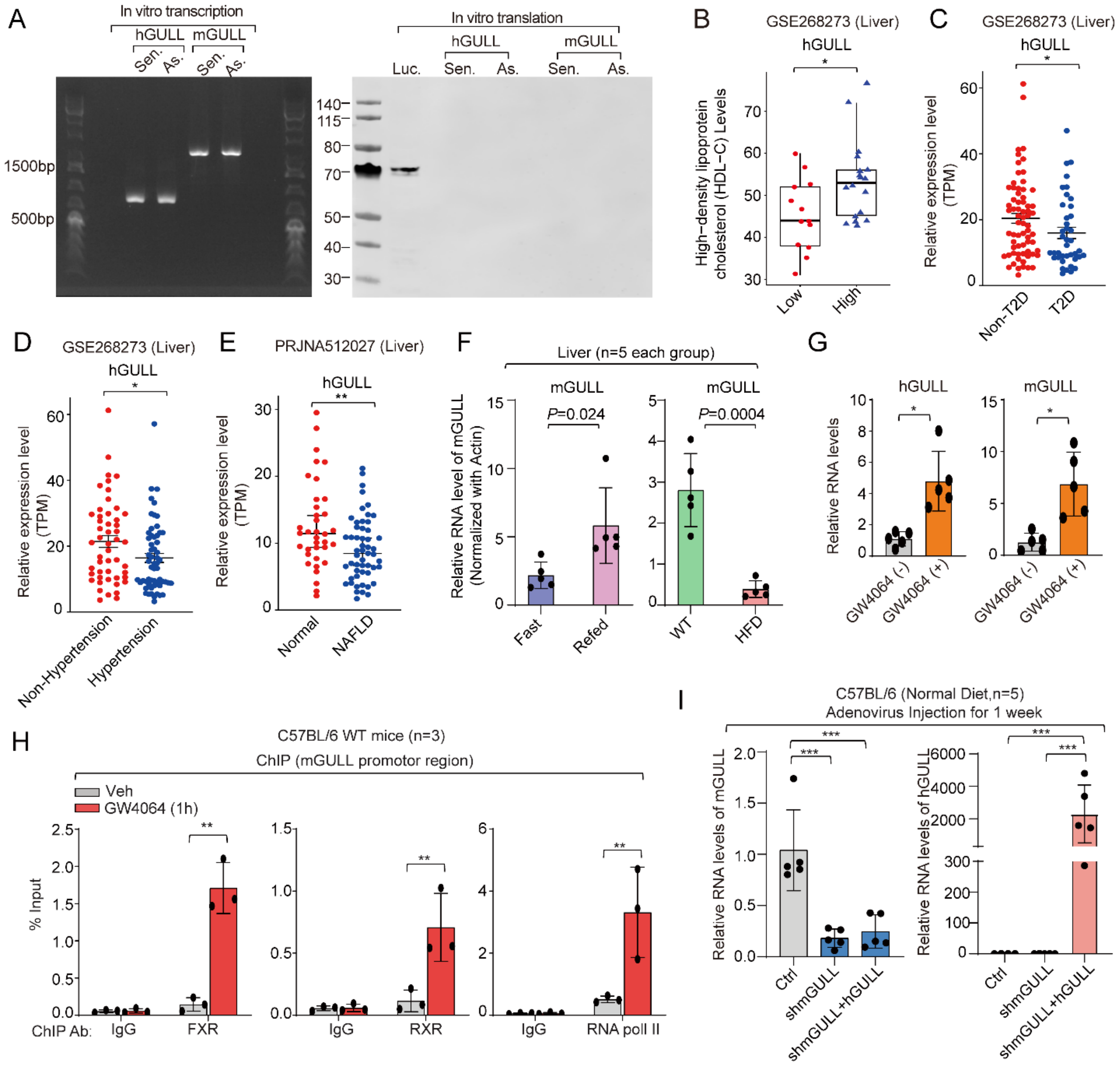

Figure S1. Characterization of hGULL/mGULL.

(A) In vitro transcription and translation assay of full-length hGULL/mGULL. Luciferase (Luc) is used as a positive control. (B) Boxplot of high-density lipoprotein cholesterol (HDL-C) levels in human populations with high or low hGULL expression. Human liver RNA-Seq data were obtained from the GEO database (GSE268273), and hGULL expression level was categorized into high (top quartile) and low (bottom quartile) groups among non-T2D individuals. *$p < 0.05$, Wilcoxon rank-sum test. (C, D) the RNA level of hGULL in the livers of normal individuals compared to patients with Type 2 Diabetes (T2D) or hypertension, based on data from GSE268273.

Results are presented as mean ± SEM, *p < 0.05, Wilcoxon rank-sum test. (E) the RNA level of hGULL in the livers of normal individuals compared to patients with Non-Alcoholic Fatty Liver Disease (NAFLD). Data were retrieved from the BioProject database (PRJNA512027). Results are shown as mean ± SEM, **p < 0.01, Wilcoxon rank-sum test. (F) The RNA level of mGULL was detected by qPCR in mouse livers from fasting (16 hours) or fast-refeeding (10 hours fasting with 6 hours refeeding), or WT and HFD mice groups. The mice in fasting or fast-refeeding group are C57BL/6 wide-type mice. (Mean ± SD, n = 5 mice per group). (G) The RNA levels of hGULL/mGULL with or without GW4064 treatment in humanized liver tissues. ***P<0.001, Data showed mean ± SD, Two-tailed Student's t-test. (H) ChIP-qPCR detection of occupancy of FXR or RXR, and RNA poll II level on mGULL promotor, with IgG as the negative control. **P<0.01, Data showed mean ± SD, Two-way ANOVA. (I) The efficiency of mGULL knockdown or the hGULL overexpression infected with adenovirus in the WT mice for one week detected by qPCR, *P<0.05, Data showed mean ± SD, Two-tailed Student's t-test.

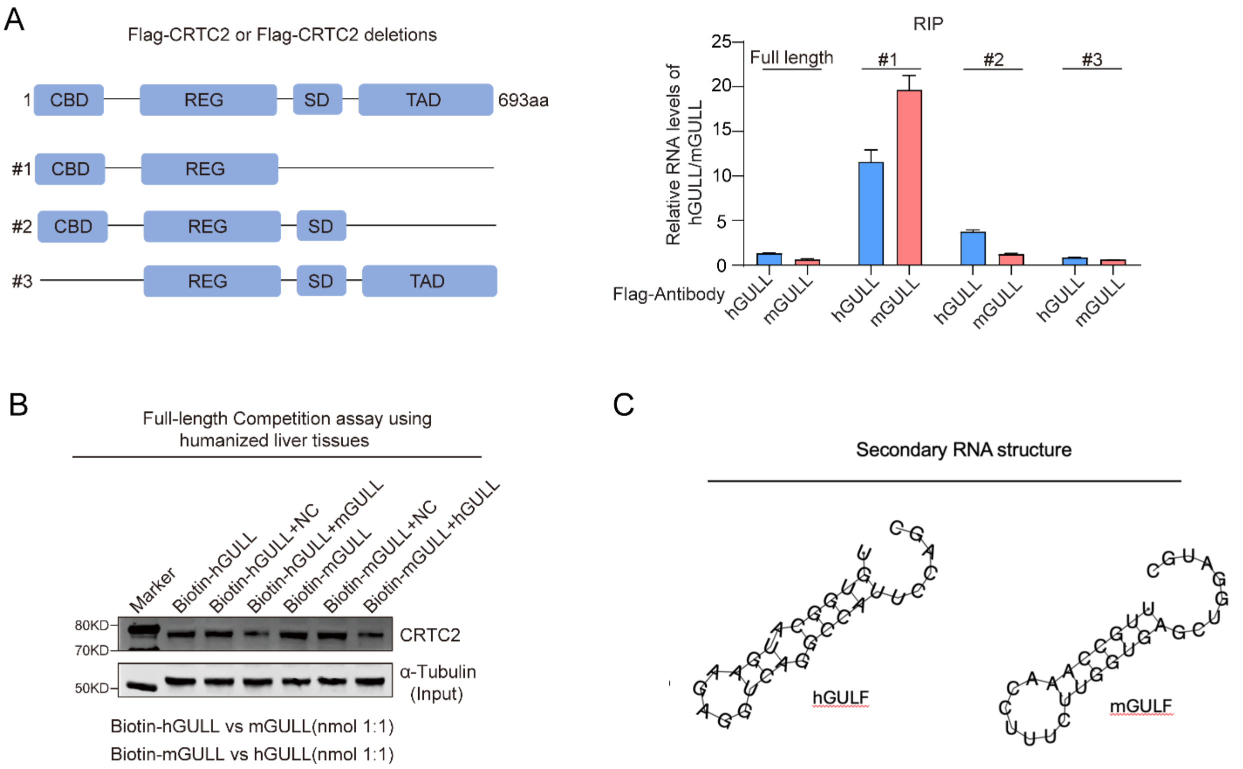

Figure S2. hGULL and mGULL are physically interacting with CRTC2.

(A) Graphic illustration shows the deletion mapping for the domains of CRTC2 and RIP analysis for hGULL/mGULL enrichment in the indicated FLAG-tagged CRTC2 full-length or truncated constructs; (B) Full-length hGULL/mGULL competition assay coupled with RNA pulldown and immune blot. Non-biotin-labeled hGULL or mGULF were used to compete with equal mole with biotin-labeled hGULL or mGULL for binding to the CRTC2; (C) Secondary structure of hGULF/mGULF predicted by RNAfold using the default settings (http://rna.tbi.univie.ac.at/cgi-bin/RNAWebSuite/RNAfold.cgi).

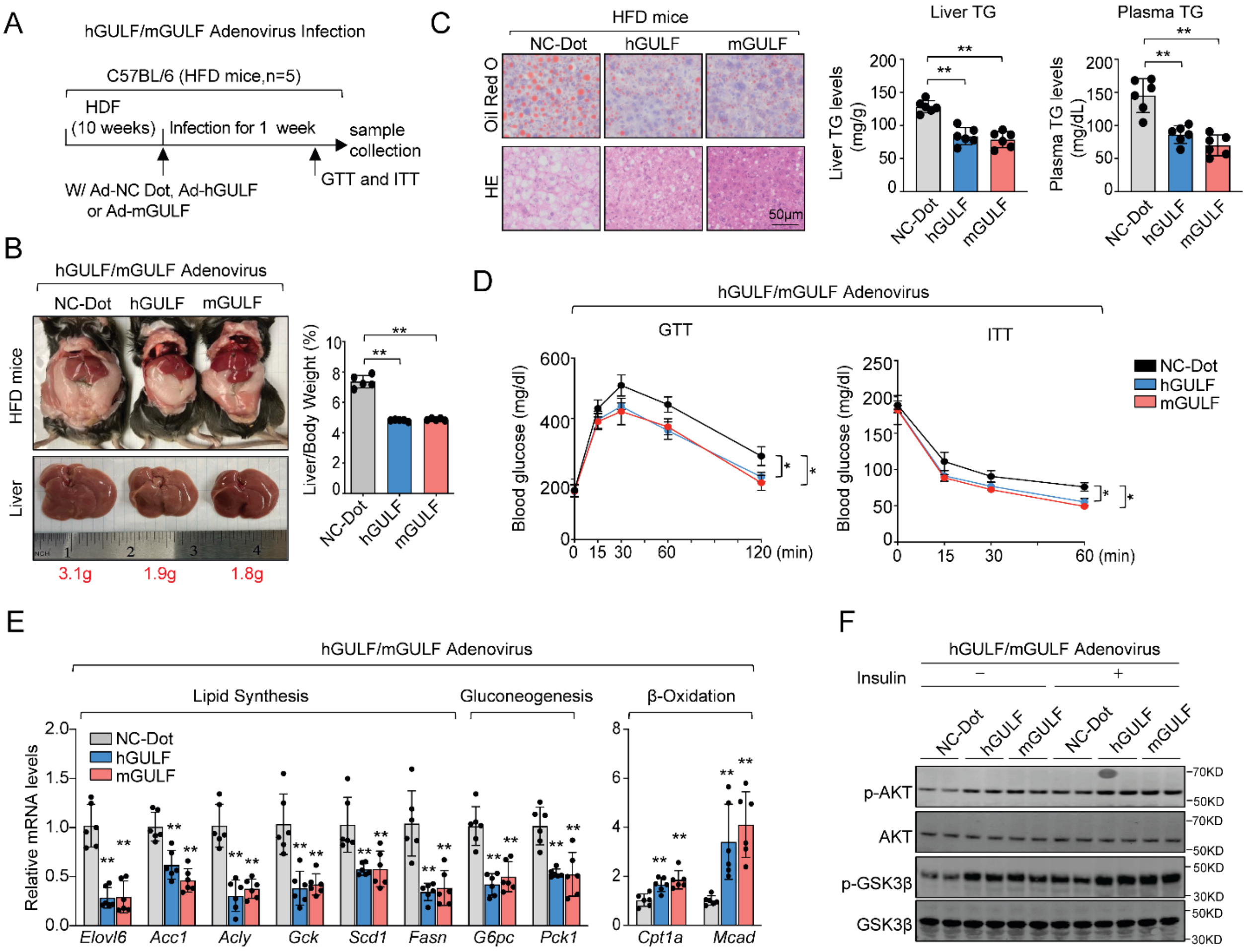

Figure S3. Adenovirus-mediated hGULF/mGULF overexpression showed strong benefits in obese mice

(A) Graphic representation of hGULF/mGULF adenovirus infection model with obese mice; (B-C) Representative images of liver and liver/body weight ratio analysis (B), Oil Red O and H&E staining, and plasma/liver TG level analysis (C) after infected with Ad-NC, Ad-hGULF, and Ad-mGULF for one week, **P<0.01, Data showed mean ± SD, One-way ANOVA; (D) GTT and ITT tests were determined in the adenovirus-mediated hGULF/mGULF mouse model. *P<0.05, Data showed mean ± SD, One-way ANOVA; (E) The mRNA levels of lipid synthesis, gluconeogenesis, and β-Oxidation genes were quantified in the liver tissues of mice infected with Ad-NC, Ad-hGULF, and Ad-mGULF adenovirus using qPCR. **P<0.01, Data showed mean ± SD, One-way ANOVA; (F) Immunoblotting of p-AKT, AKT, p-GSK3β, and GSK3β in the Ad-NC, Ad-hGULF, and Ad-mGULF groups with or without insulin treatment.

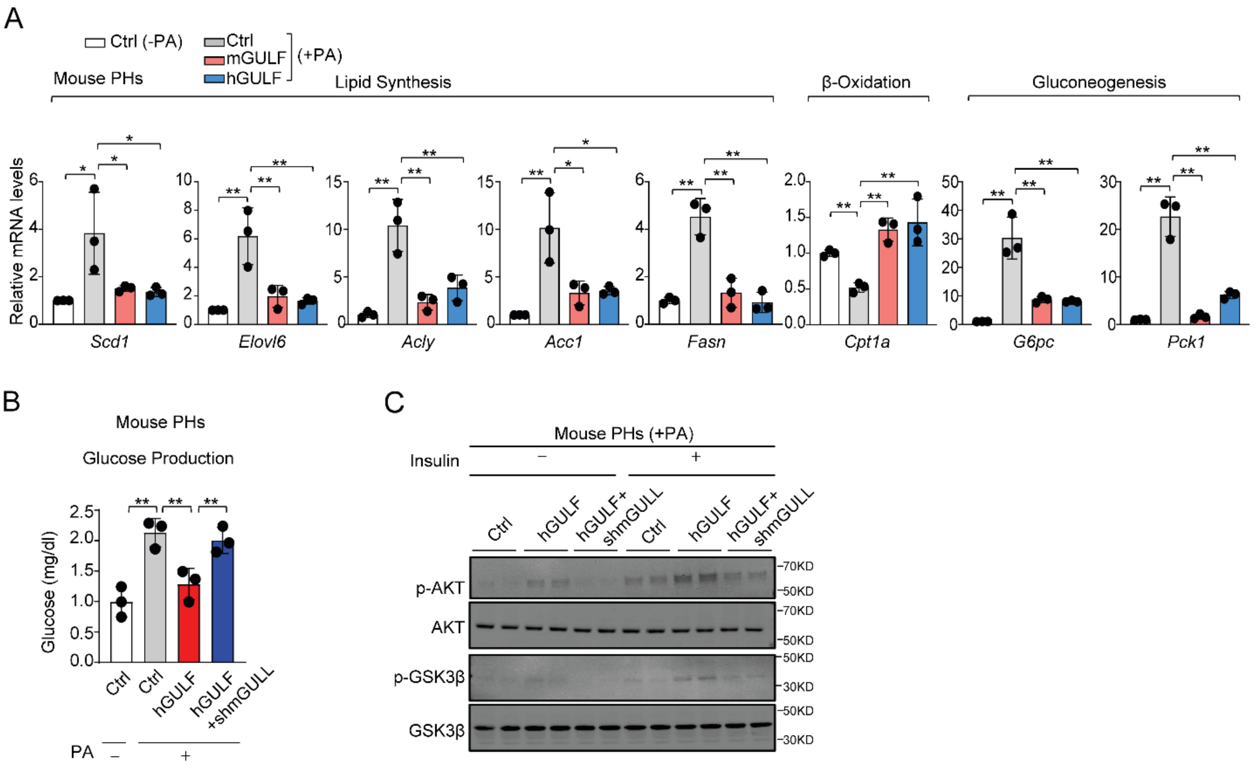

Figure S4. hGULF/mGULF regulated lipid and glucose metabolism in mouse PHs

(A) The mRNA levels of lipid synthesis, gluconeogenesis, and β-Oxidation genes were quantified in mouse PH cells after transfected with hGULF/mGULF with or without PA treatment. *P<0.05 and **P<0.01, Data showed mean ± SD, One-way ANOVA; (B) The glucose level was determined in the mouse PH cells, including the hGULF overexpression group and hGULF overexpressed accompanied by mGULL knockdown. **P<0.01, Data showed mean ± SD, Two-tailed Student's t-test; (C) Immunoblotting of p-AKT, AKT, p-GSK3β, and GSK3β in the Ctrl, hGULF overexpression, and hGULF overexpressed accompanied by mGULL knockdown groups with or without insulin treatment.

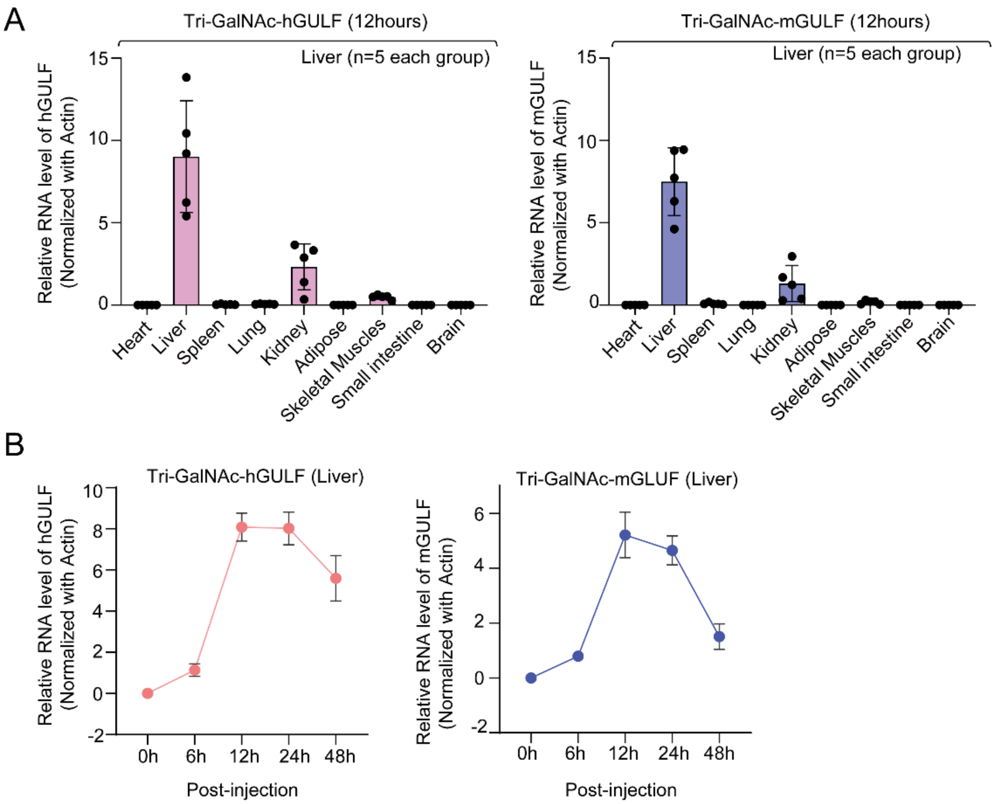

Figure S5. The distribution of Tri-GalNAc-hGULF/mGULF in mouse liver.

(A) The relative RNA level indicated the distribution of h/mGULF in various mice organs. (Mean ± SD, n = 5 mice per time point). (B) The RNA level of h/mGULF was detected by qPCR in mouse livers at the indicated time points post-I.V. administration (0.01 mg/g, single dose, Mean ± SD, n = 3 mice per time point). Data are presented as mean ± SD and analyzed using a two-tailed Student's t-test.

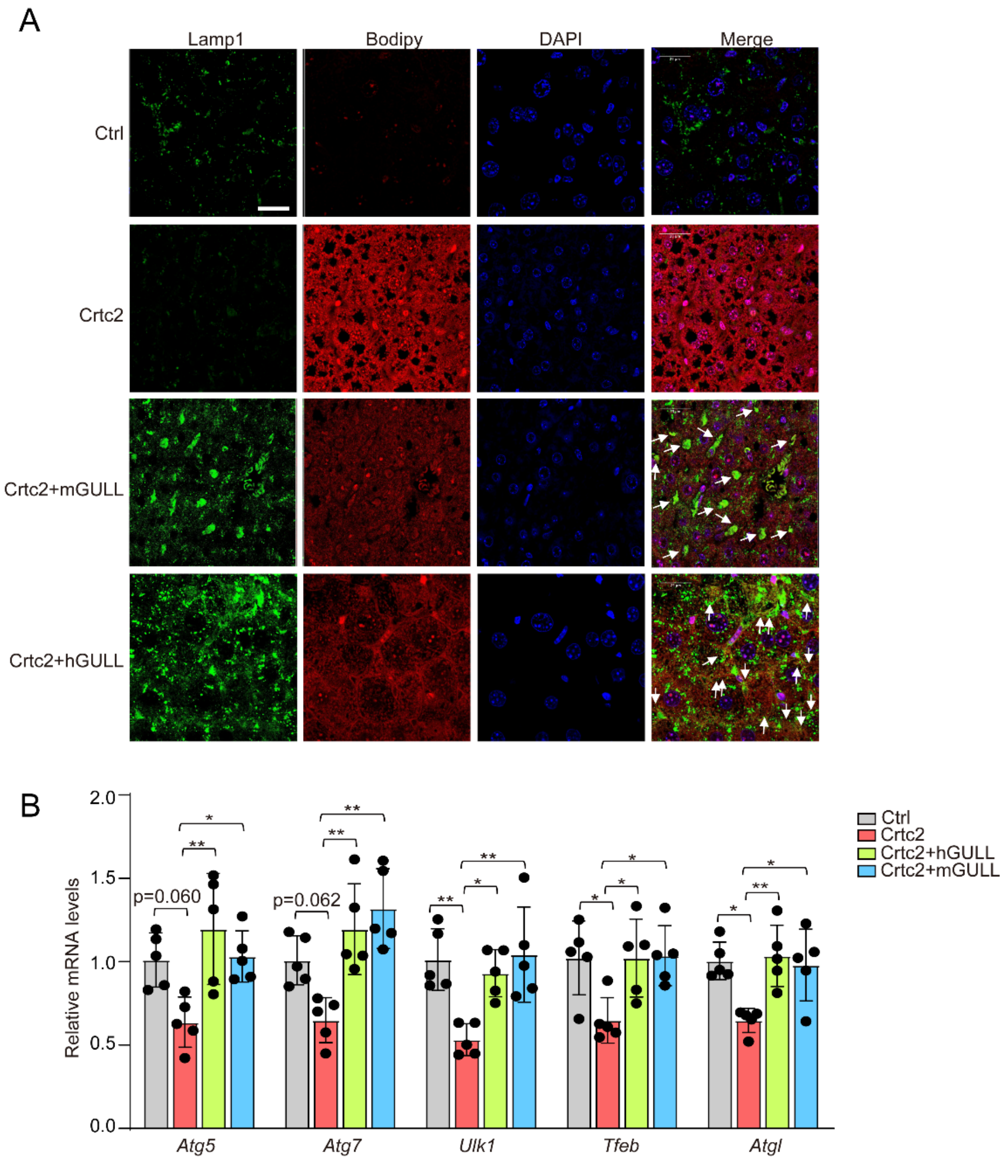

Figure S6. hGULF/mGULF-Crtc2 are involved in lipophagy in mouse liver.

(A) For in vivo detection of hepatic lipophagy, paraffin-embedded liver slides were incubated overnight with an anti-LAMP1 antibody, followed by secondary antibody, Alexa 488 donkey anti-rabbit for 1 h. Lipids were stained using a final concentration of 1 μg/ml of BODIPY 581/591 for 10 min. Nuclei were labeled with DAPI and images were taken and analyzed by confocal microscopy (Zeiss, LSM880). White arrows indicate the area where LAMP1 and BODIPY co-localization, representing lipophagy. (B) The mRNA levels of lipophagy (autophagy) genes were quantified in Ctrl, Crtc2 overexpression, and Crtc2+hGULL or mGULL overexpression groups using qPCR, *P<0.05, **P<0.01, Data showed mean ± SD, One-way ANOVA.

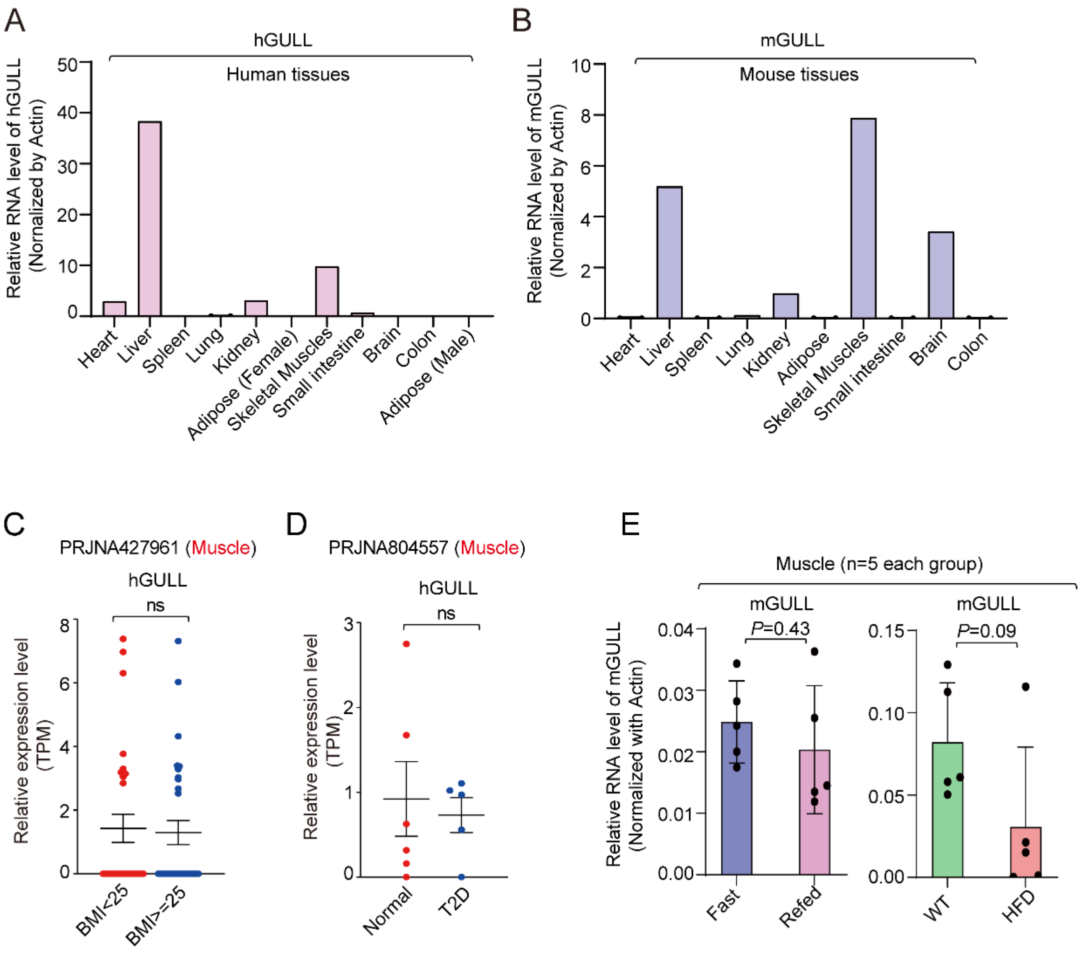

Figure S7. The distribution of hGULF/mGULF in human or mouse organs.

(A, B) The distribution of h/mGULL in various human and mouse organs was assessed using a commercial cDNA panel by qPCR, with Actin as the internal control. (C) The relative expression level of hGULL in the muscles of patients with BMI < 25 or BMI ⩾ 25. Data were retrieved from the BioProject database (PRJNA427961). Results are shown as mean ± SEM, "ns" stands for "not significant", Wilcox rank-sum test). (D) The relative expression level of hGULL in the muscles of normal individuals compared to T2D patients. Data were retrieved from the BioProject database (PRJNA804557). Results are shown as mean ± SEM, Wilcox rank-sum test). (E) The RNA level of mGULL was detected by qPCR in mouse muscles from fasting, fast-refeeding, or WT and HFD groups (Mean ± SD, n = 5 mice per group).

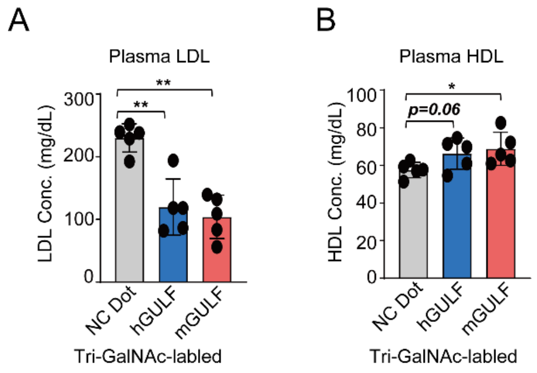

Figure S8. The toxicity of Tri-GalNAc-hGULF/mGULF in mouse liver.

(A, B) The plasma LDL and HDL cholesterol levels were measured in mice injected with Tri-GalNAc-NC, Tri-GalNAc-hGULF, or Tri-GalNAc-mGULF oligos every two days for two weeks. $^*P < 0.05$, $^{**}P < 0.01$; Data are presented as mean ± SD and analyzed using a two-tailed Student's t-test.
