# Supplementary material for: The functionally conserved human lncRNA motif GULF lowers glucose and lipid levels in obese mice
Source: J Clin Invest. 2025 Sep 16;135(18):e186355. doi: 10.1172/JCI186355 (PMC12435843; doi:10.1172/JCI186355)

Figure 1I

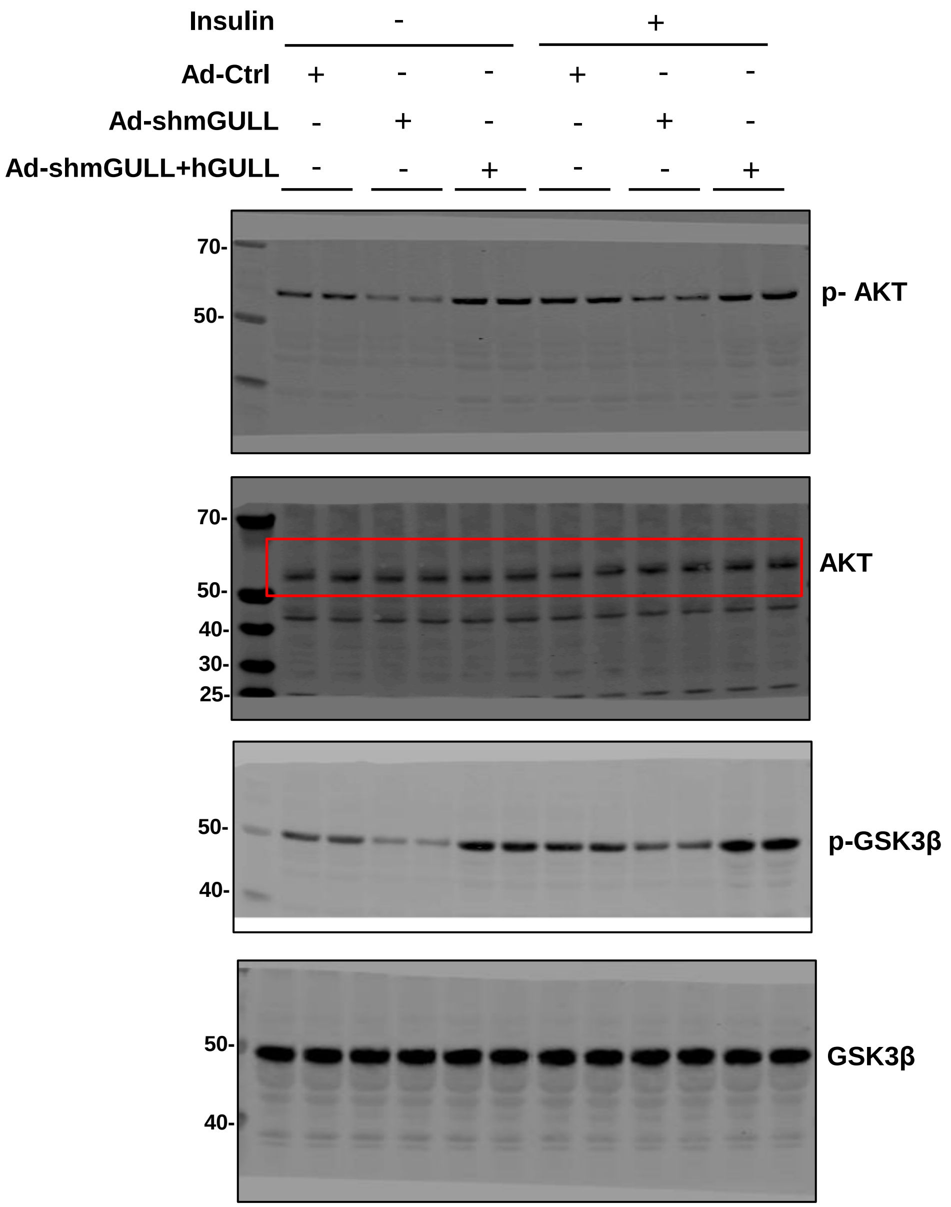

Figure 2A

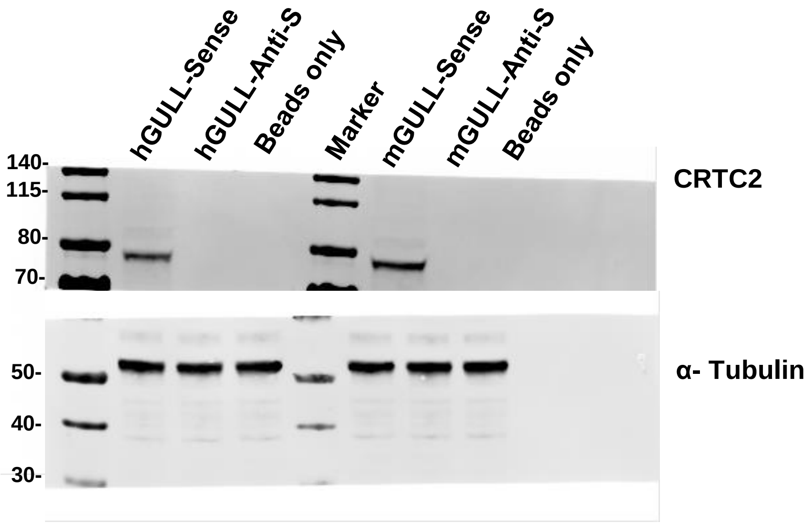

Figure 3A

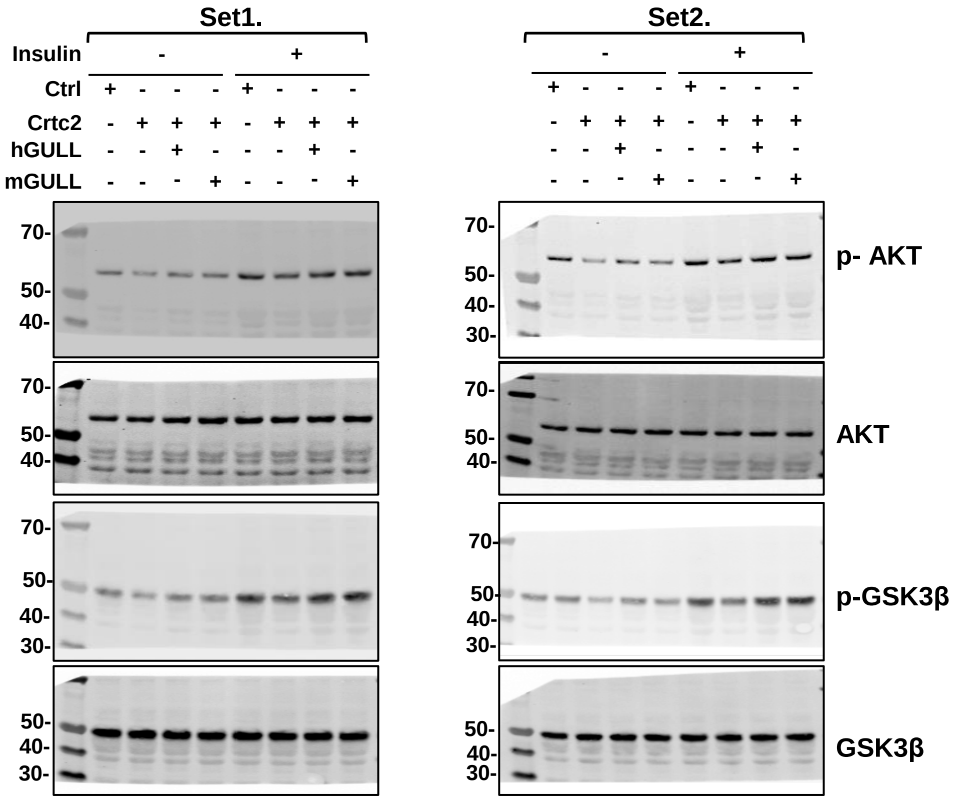

Figure 3B

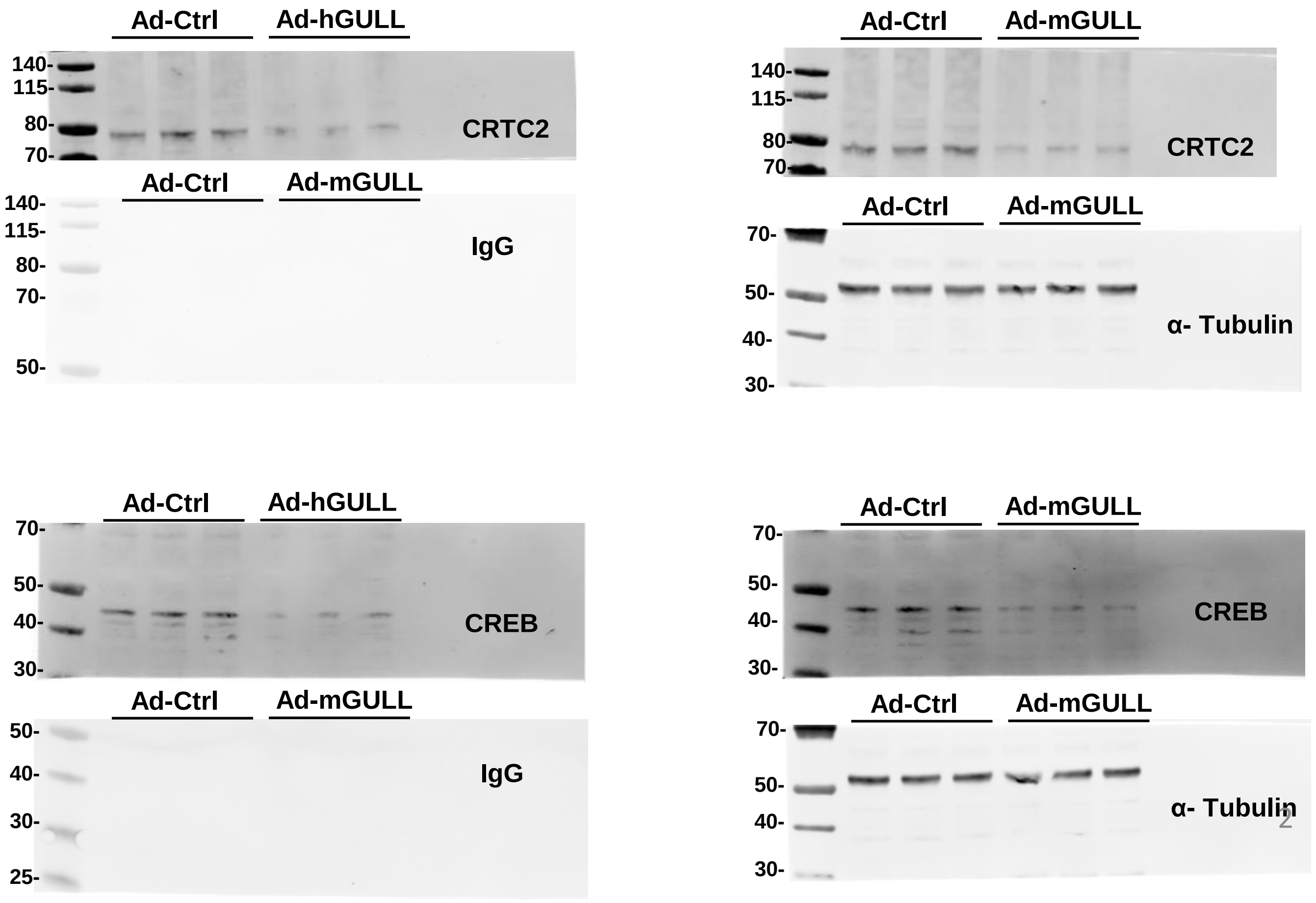

Figure 6A

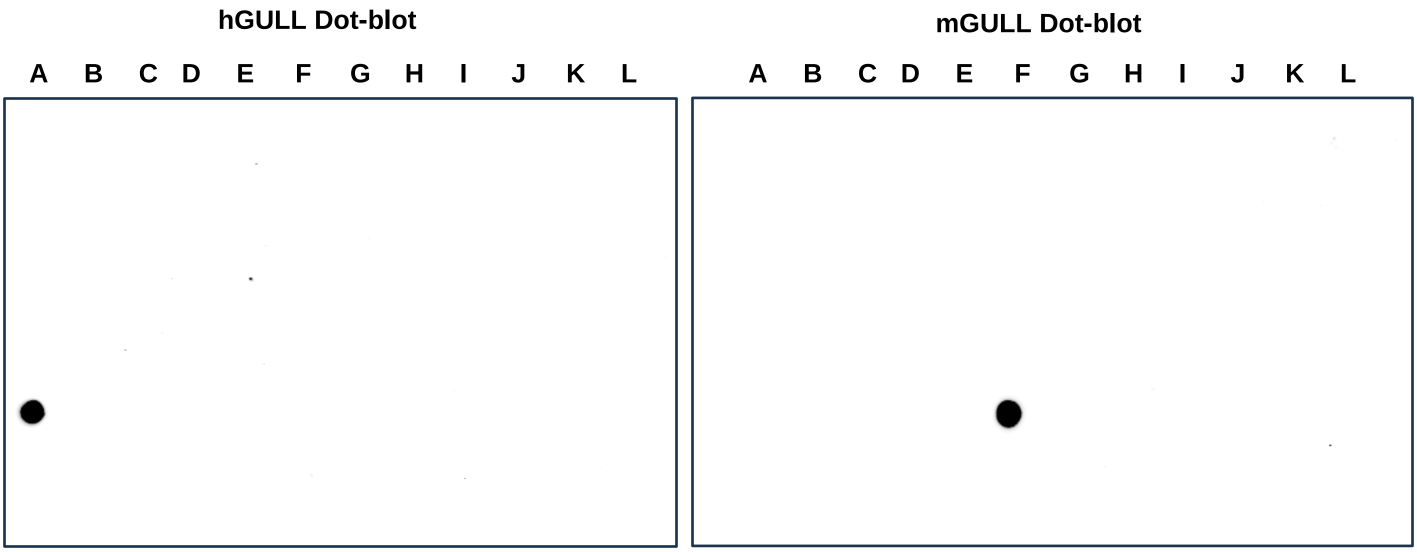

Figure 6B

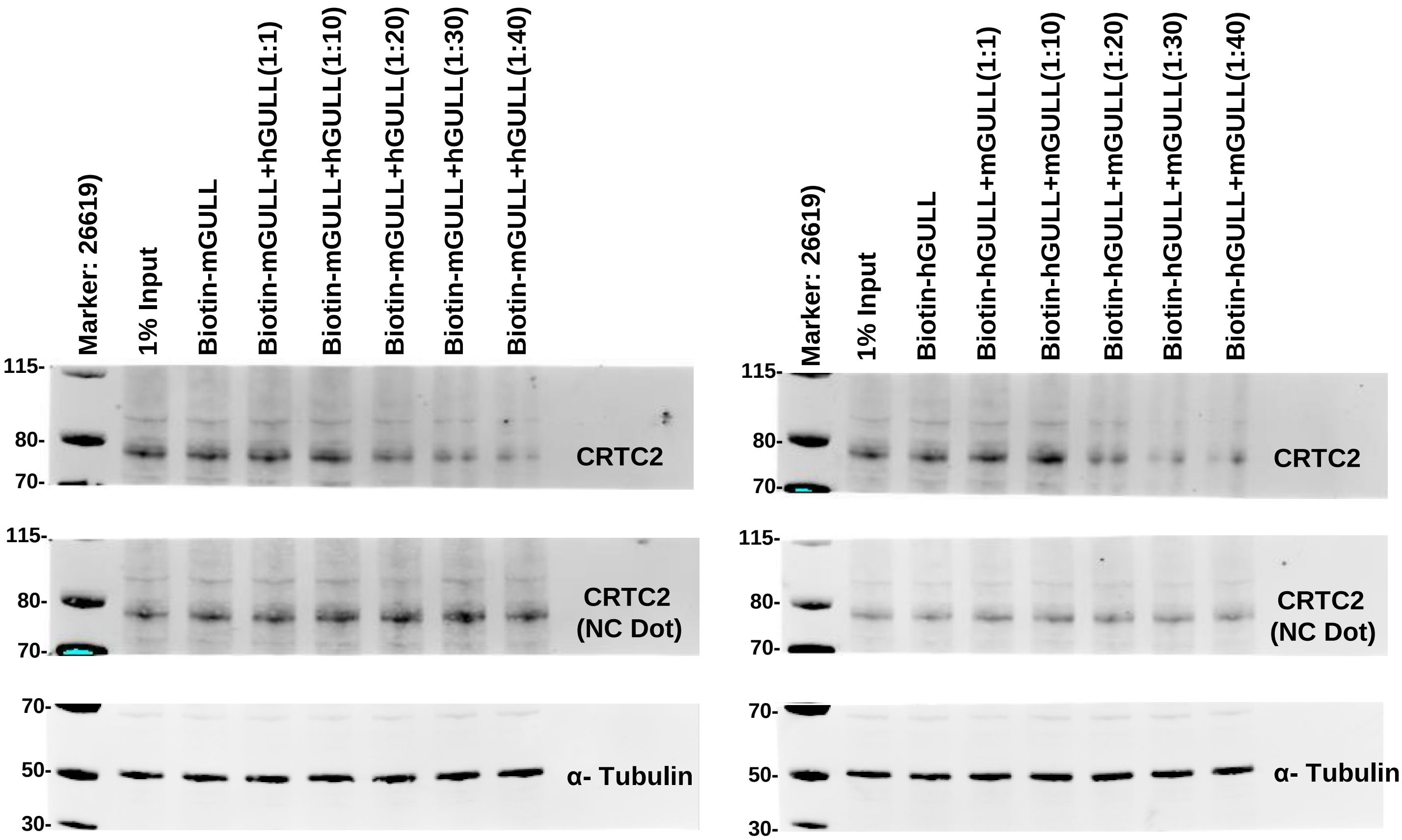

Figure 7A

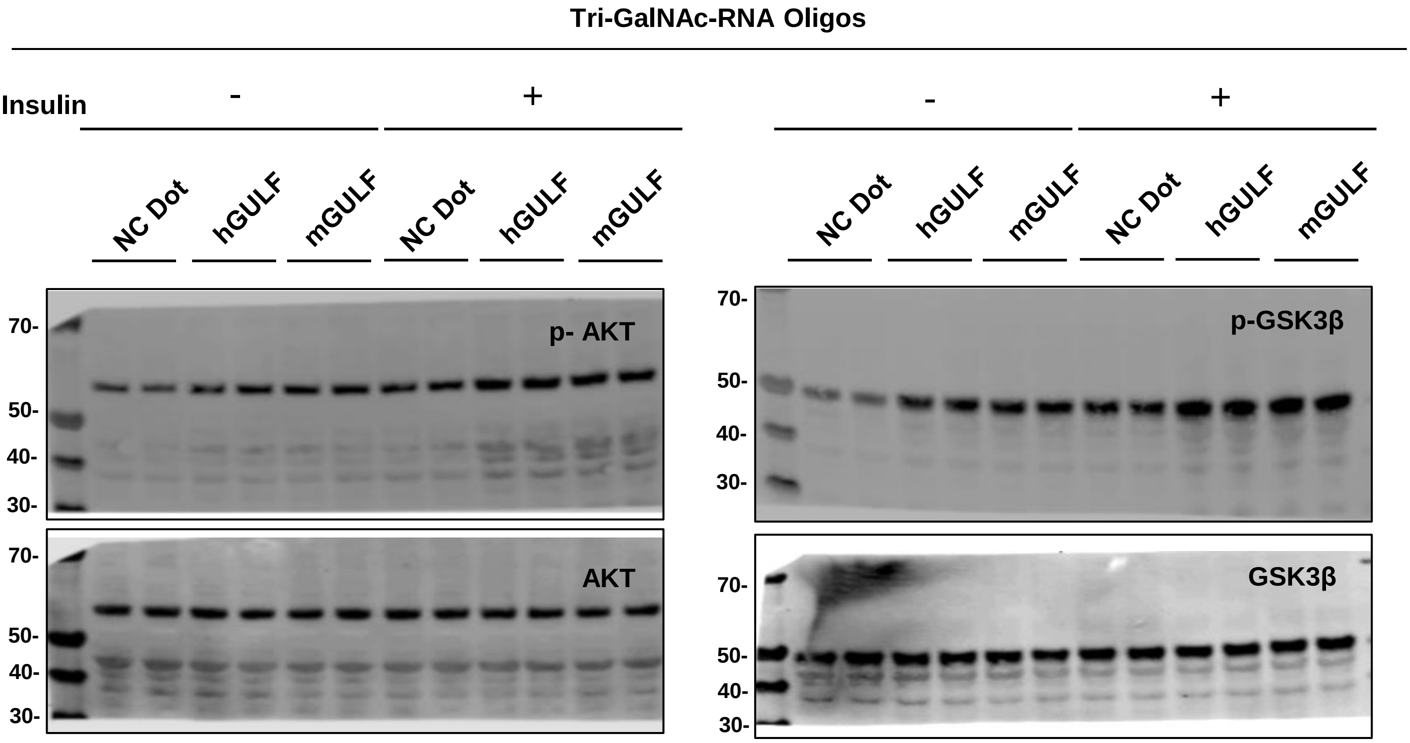

Figure 7B

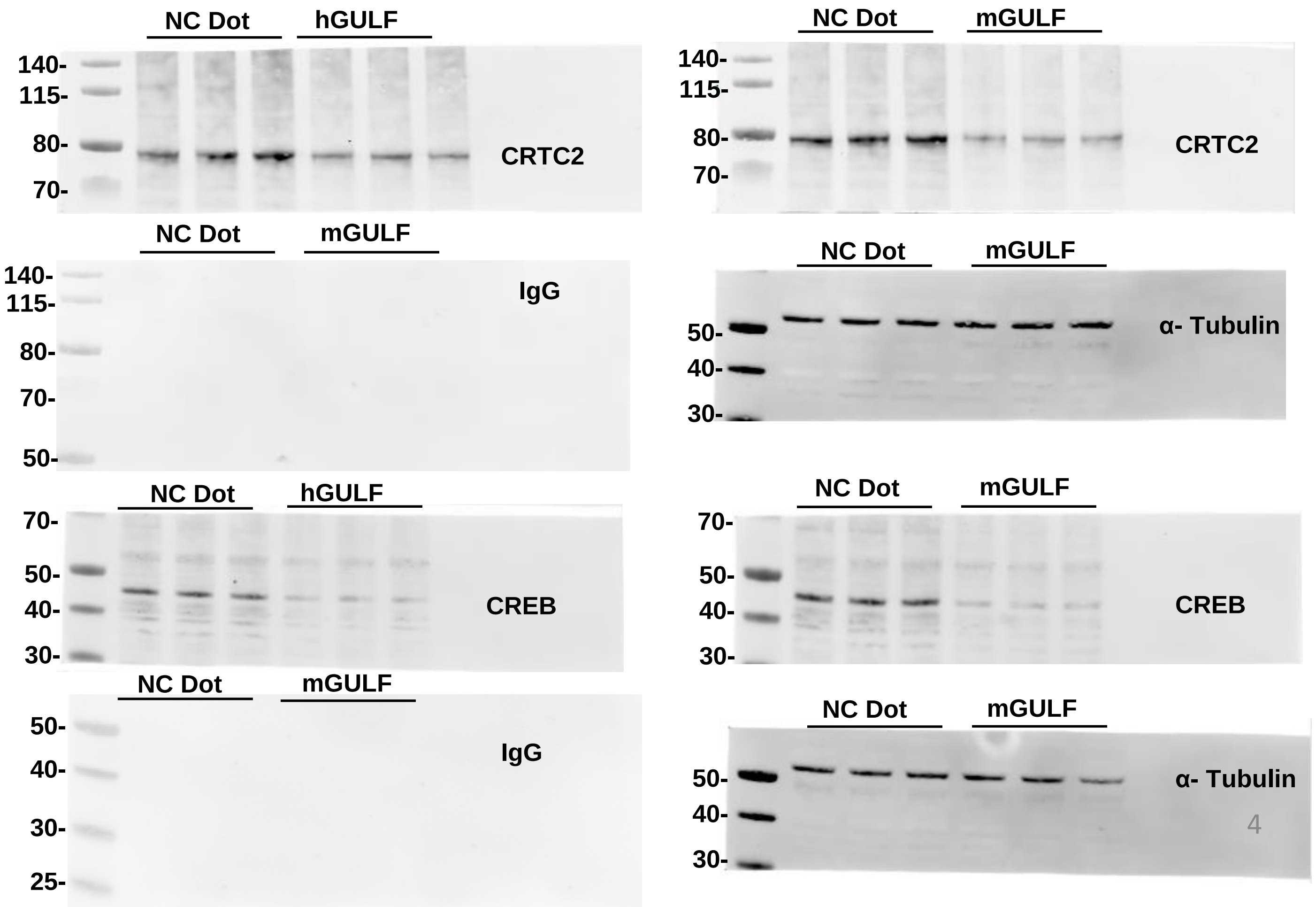

Figure S1A

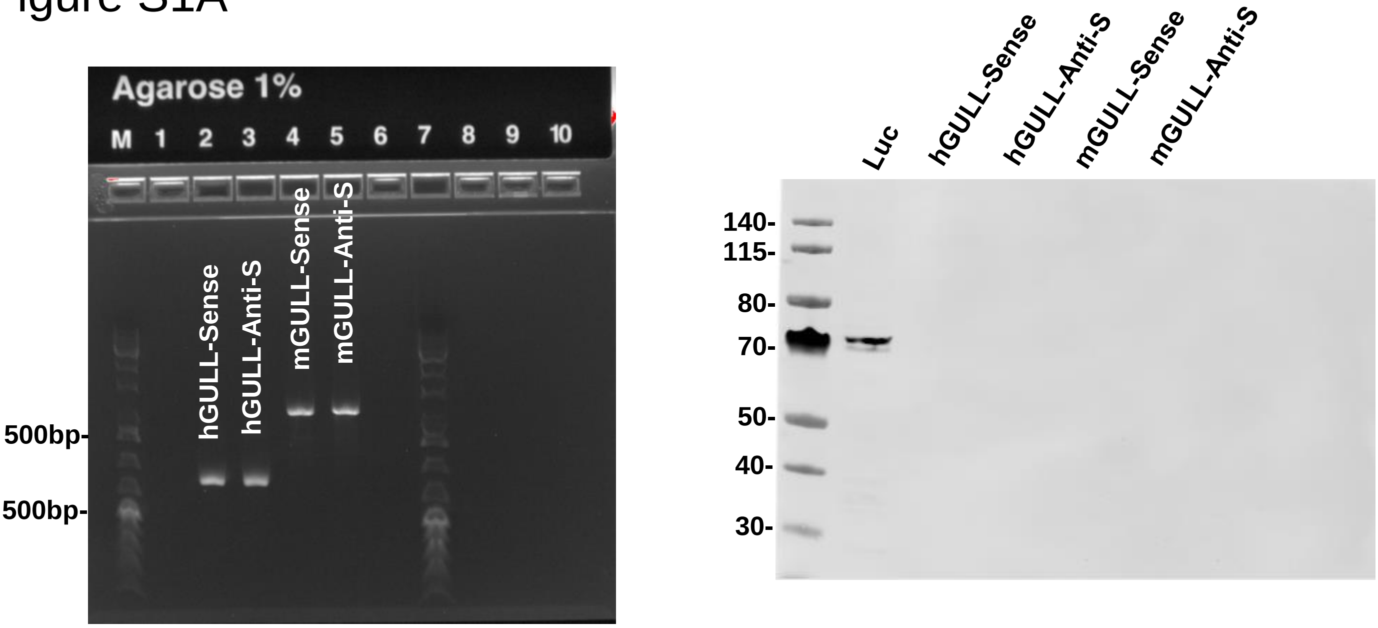

Figure S2B

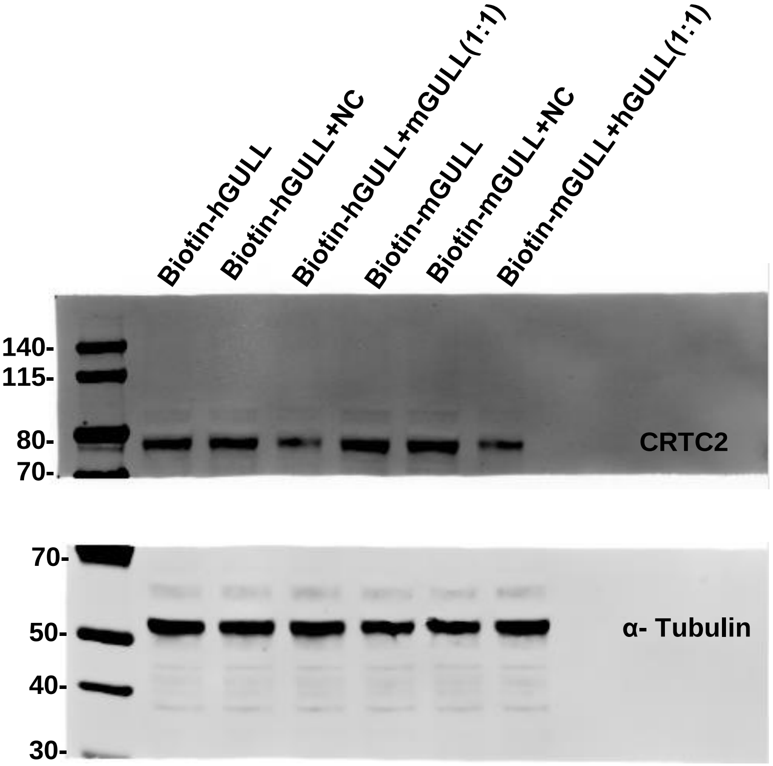

Figure S3F

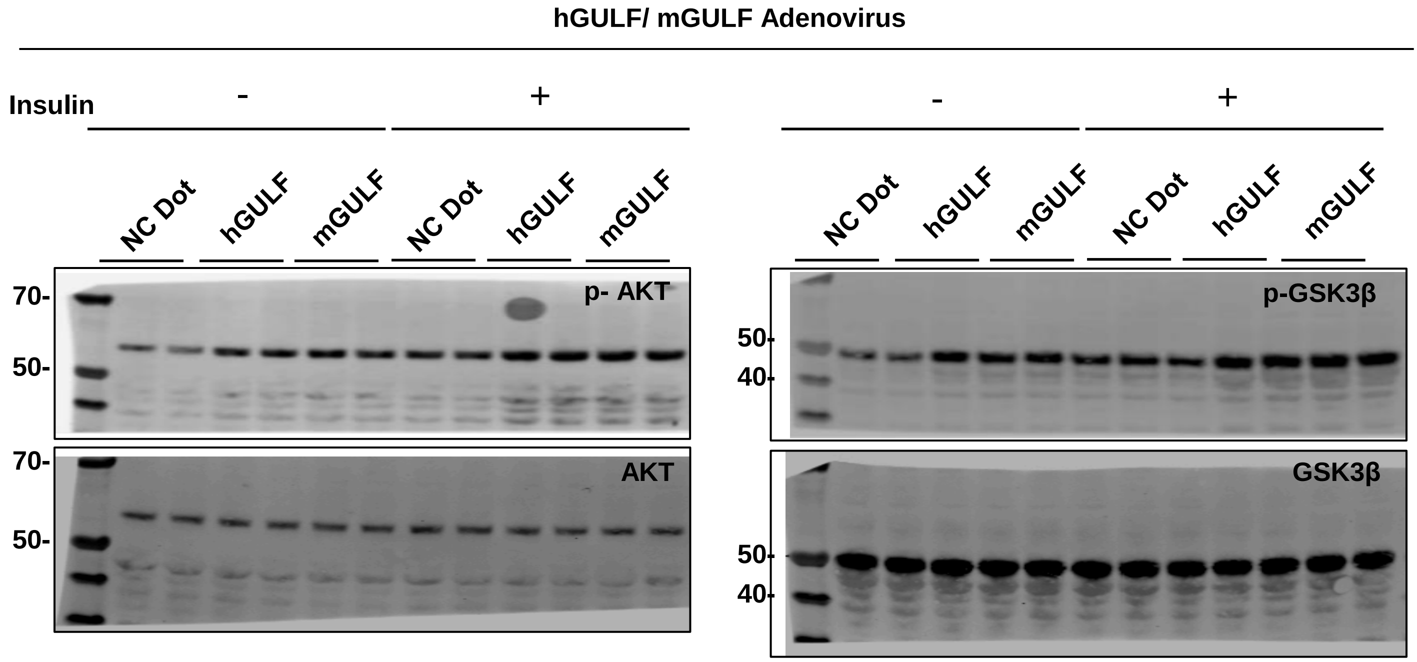

Figure S4C

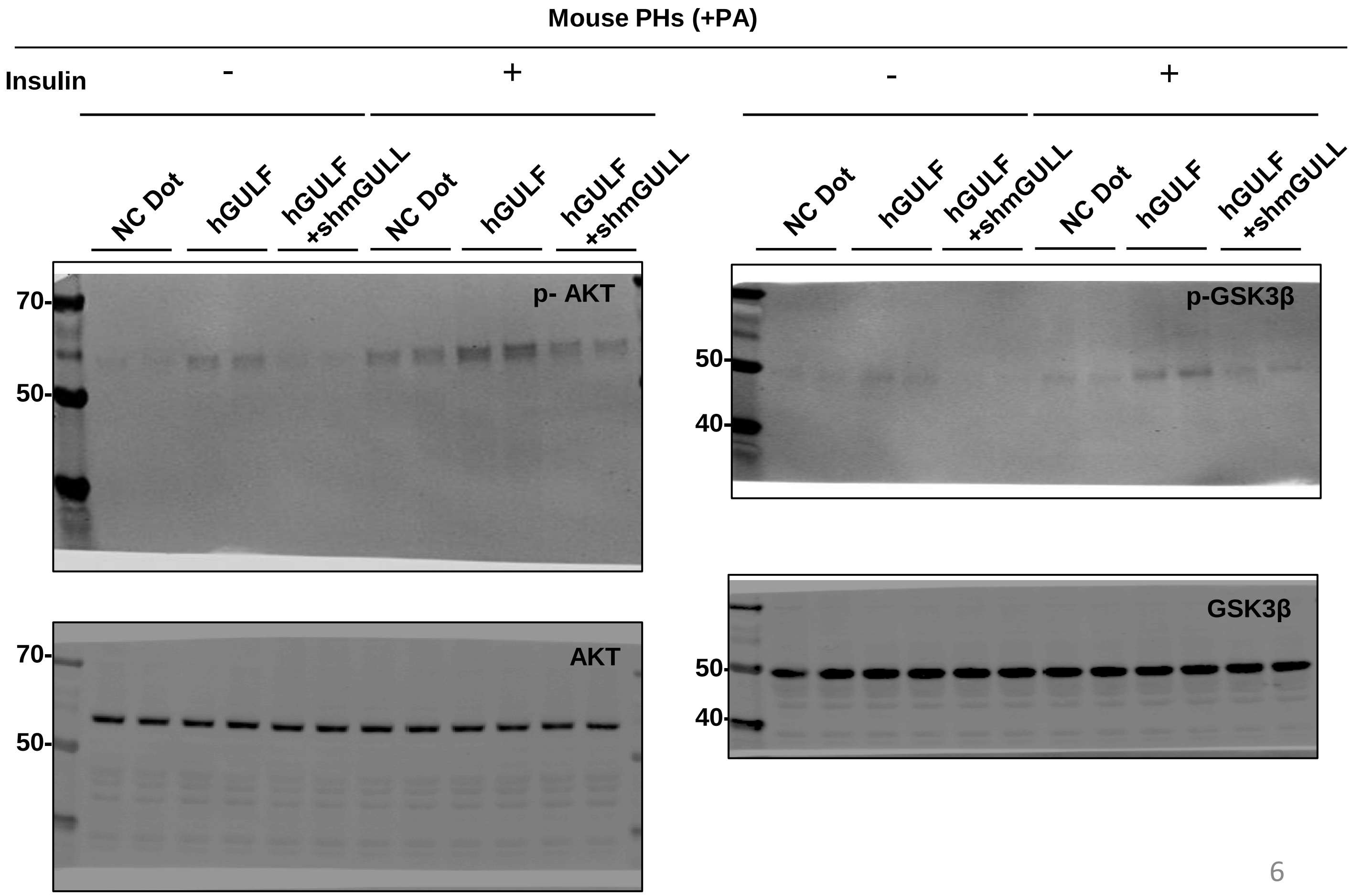

Supplement: Unedited blot and gel images [file jci-135-186355-s157.pdf]
